# Supplementary material for: Effects of LPS from Rhodobacter sphaeroides, a Purple Non-Sulfur Bacterium (PNSB), on the Gene Expression of Rice Root
Source: Microorganisms. 2023 Jun 28;11(7):1676. doi: 10.3390/microorganisms11071676 (PMC10383378; doi:10.3390/microorganisms11071676)
Supplement: Supplementary file 1 [file microorganisms-11-01676-s001.zip › Figures S1-S6.pdf]

Figure S1a

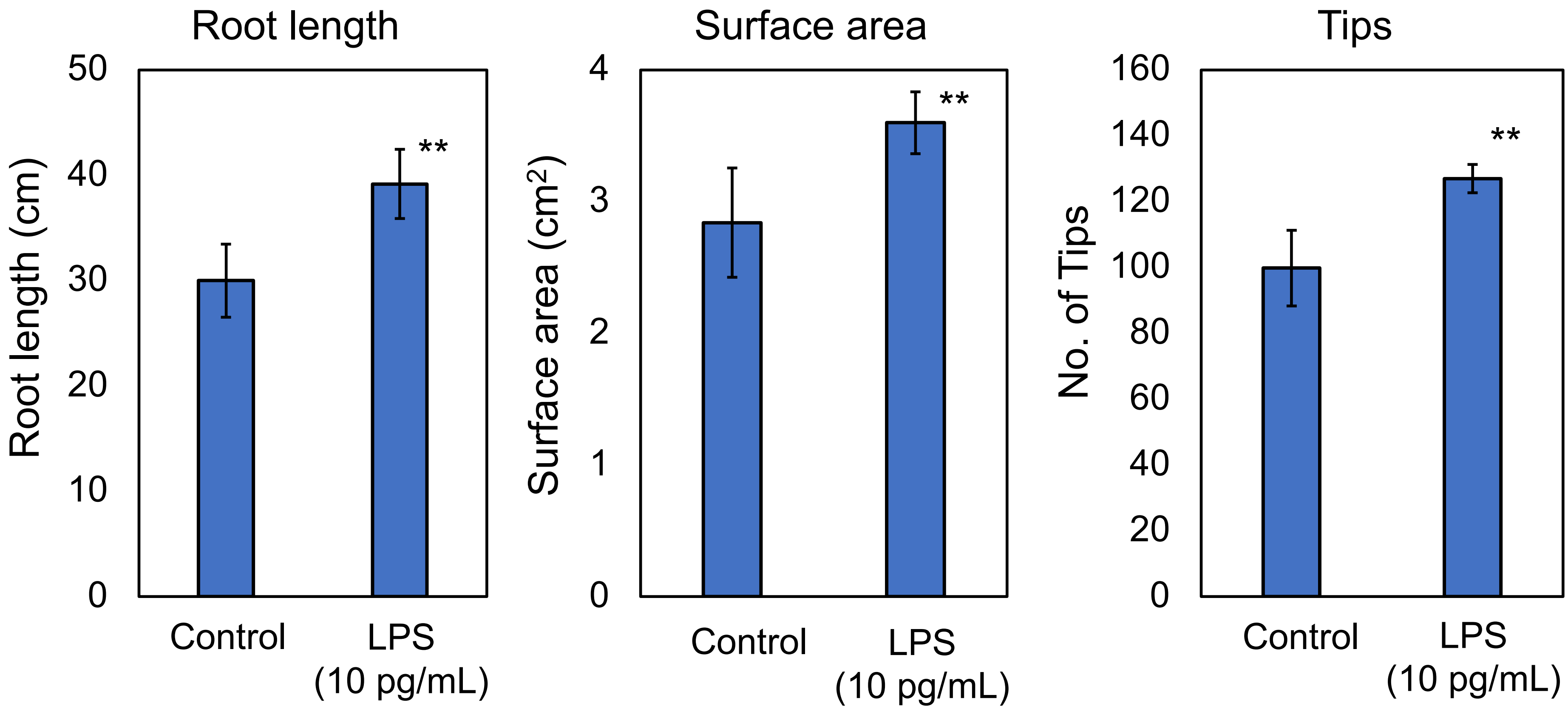

Figure S1a Effects of LPS of *Rhodobacter sphaeroides* NBRC 12203 on root development of rice seedling.

Rice seeds were aseptically germinated on 0.8% agar medium containing 10 pg/mL LPS in 15 mL test tube, and grown for 7 days. The root development was evaluated by total root, root surface area and number of root tips using WinRhizo imaging analyses.

Data are means $\pm$ SD (n = 5). Asterisk indicates the result that was significantly different (**$p<0.01$) from the control.

Figure S1b

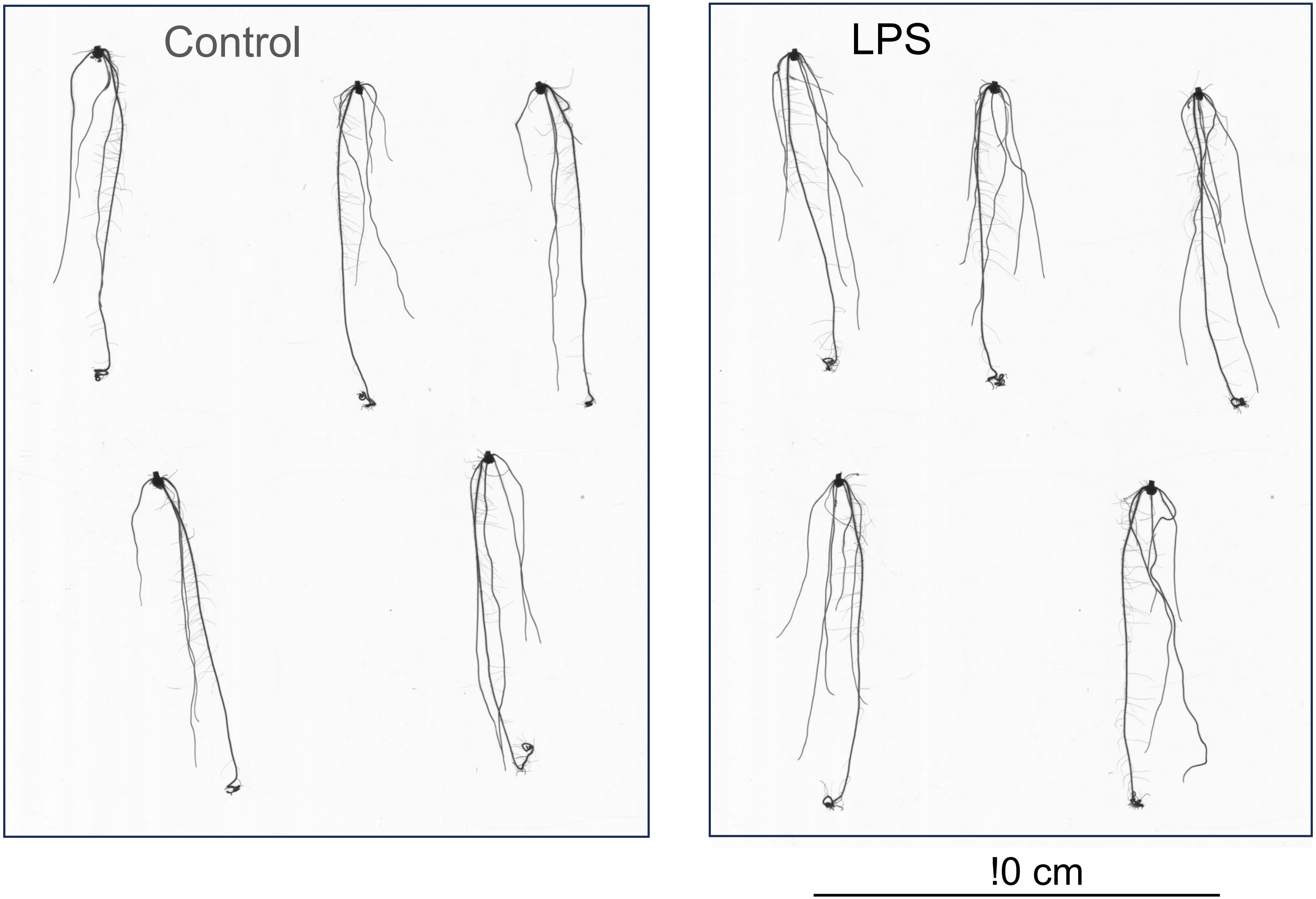

Figure S1b Scan images of the roots of seedlings.
Roots of seedlings were floated in water and scanned using a scanner (Epson GT-X970, Epson Co. Ltd., Japan).

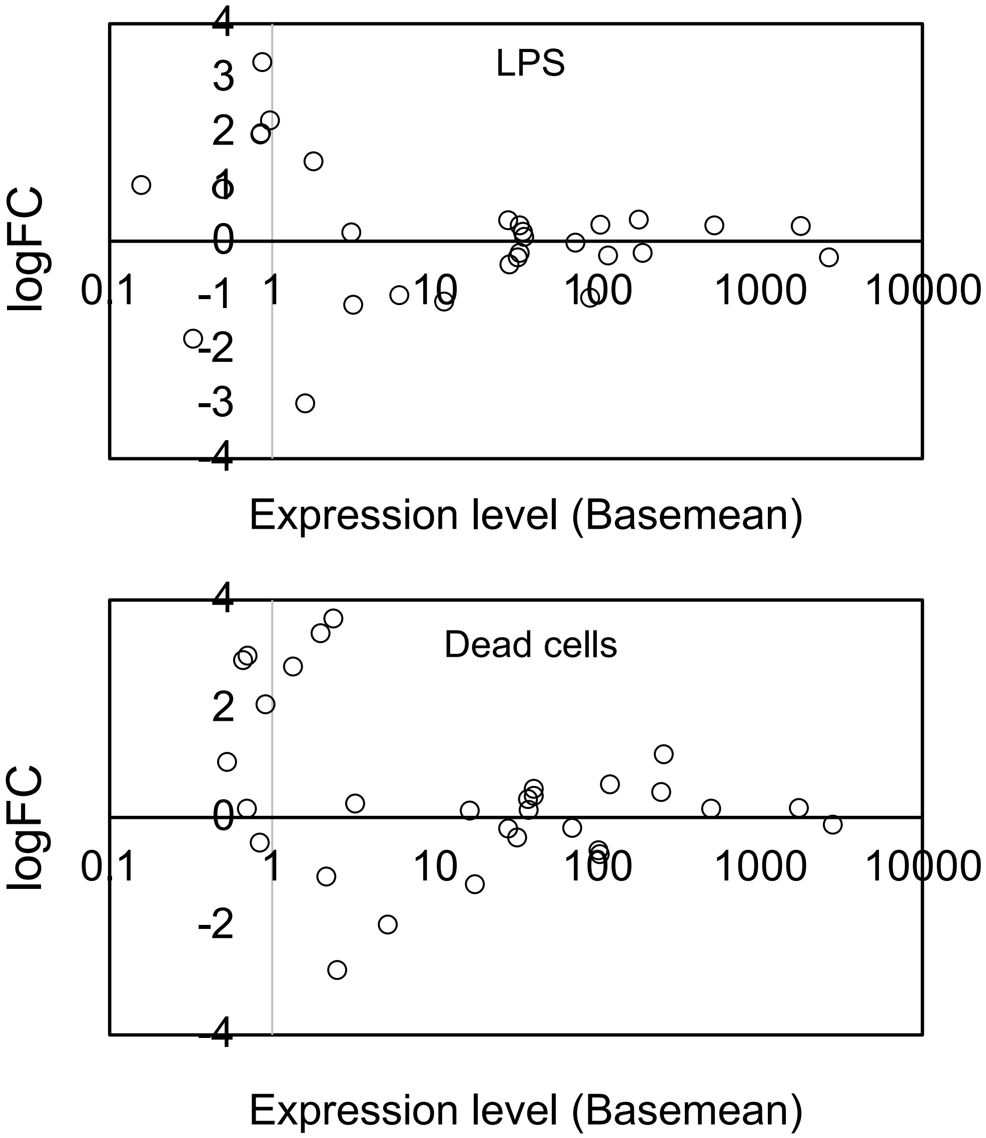

Figure S2 Plot of the logFC values and expression level (baseMean) of 30 terpenoid biosynthesis genes.

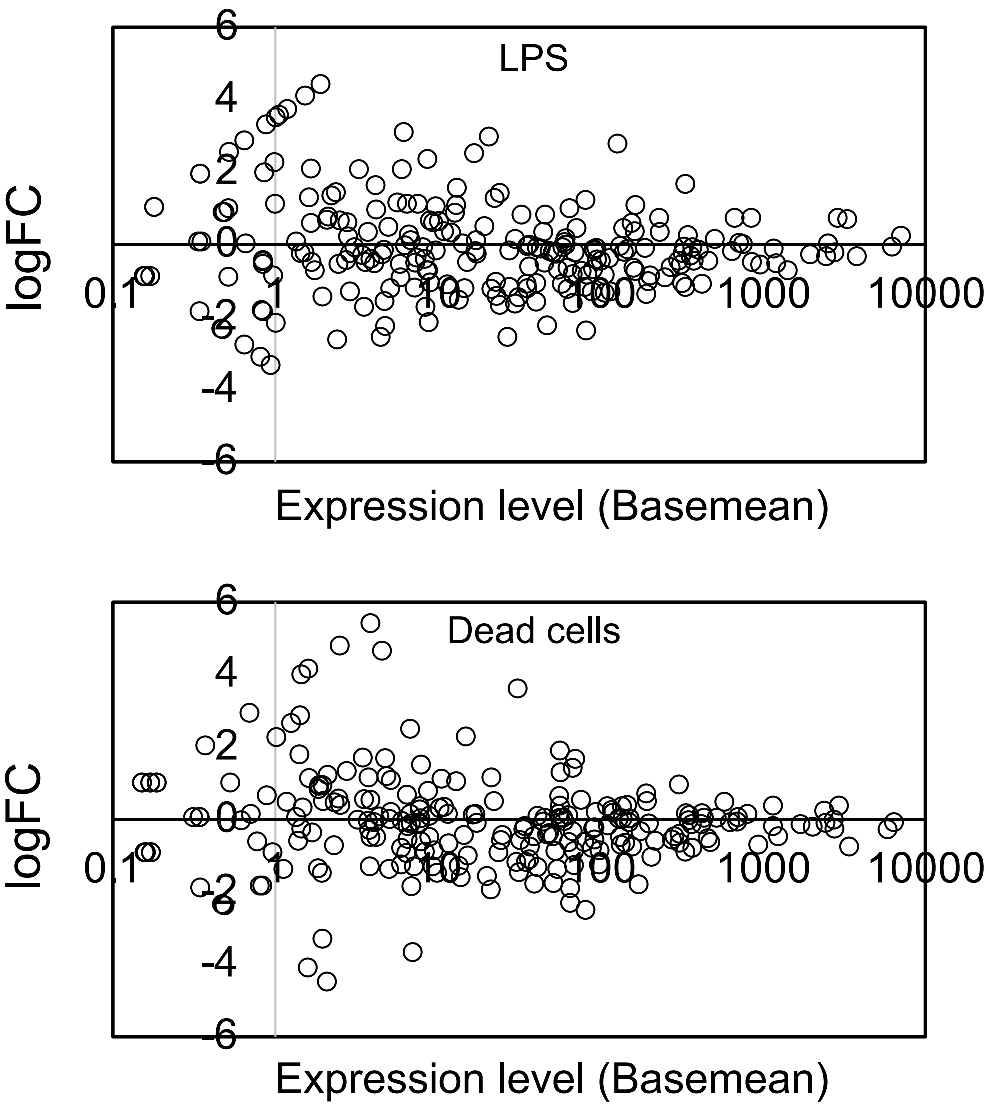

Figure S3 Plot of the logFC values and expression level (baseMean) of 233 CYP genes.

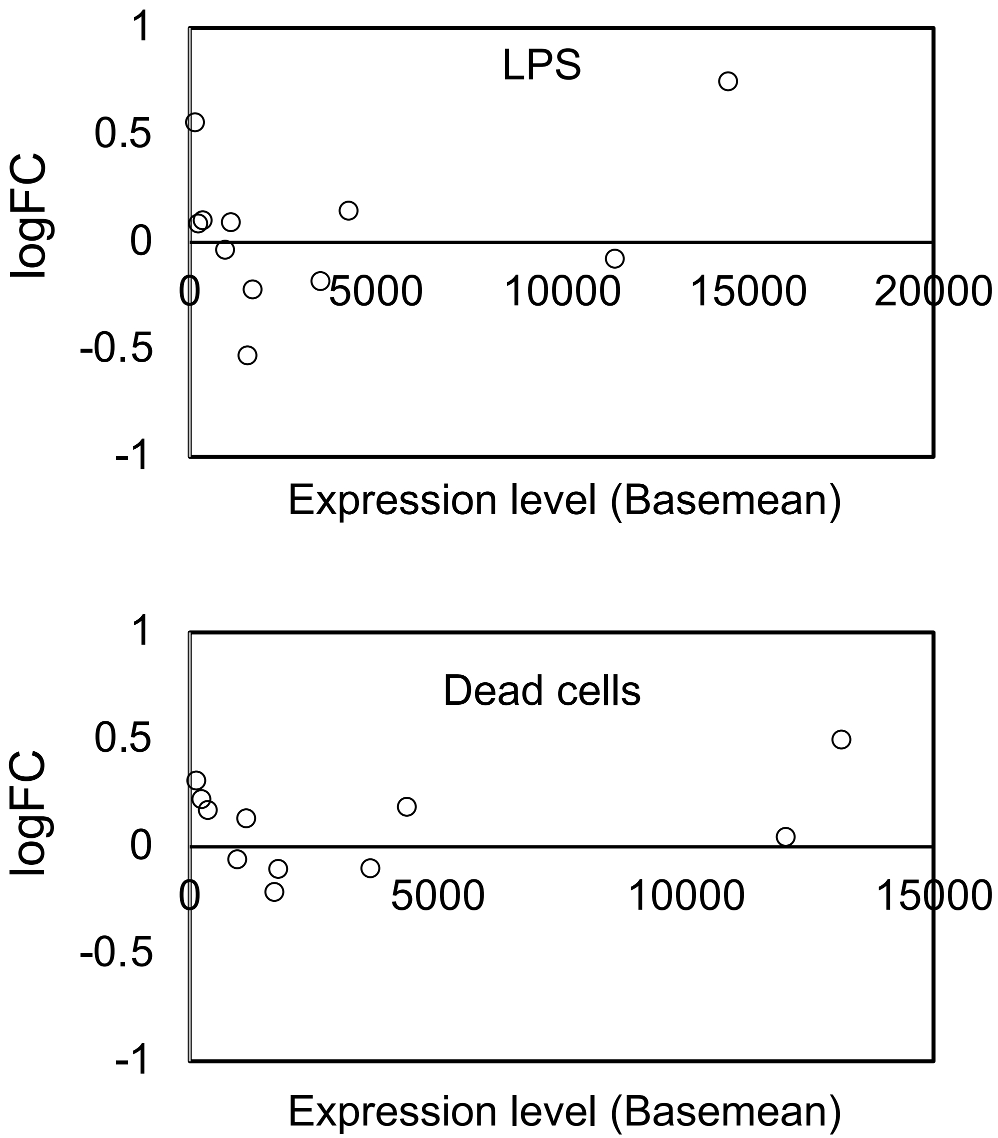

Figure S4 Plot of the logFC values and expression level (baseMean) of 11 SOD genes.

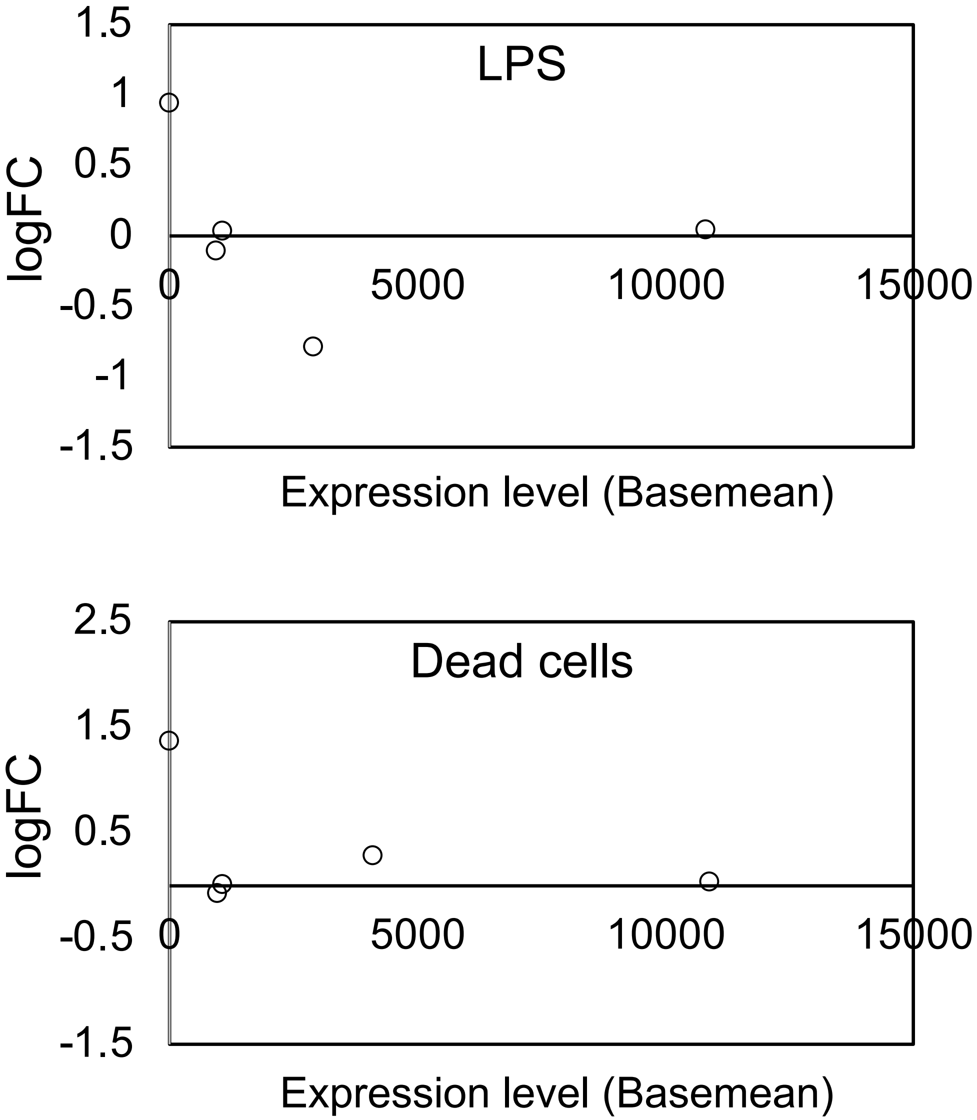

Figure S5 Plot of the logFC values and expression level (baseMean) of 5 catalase genes.

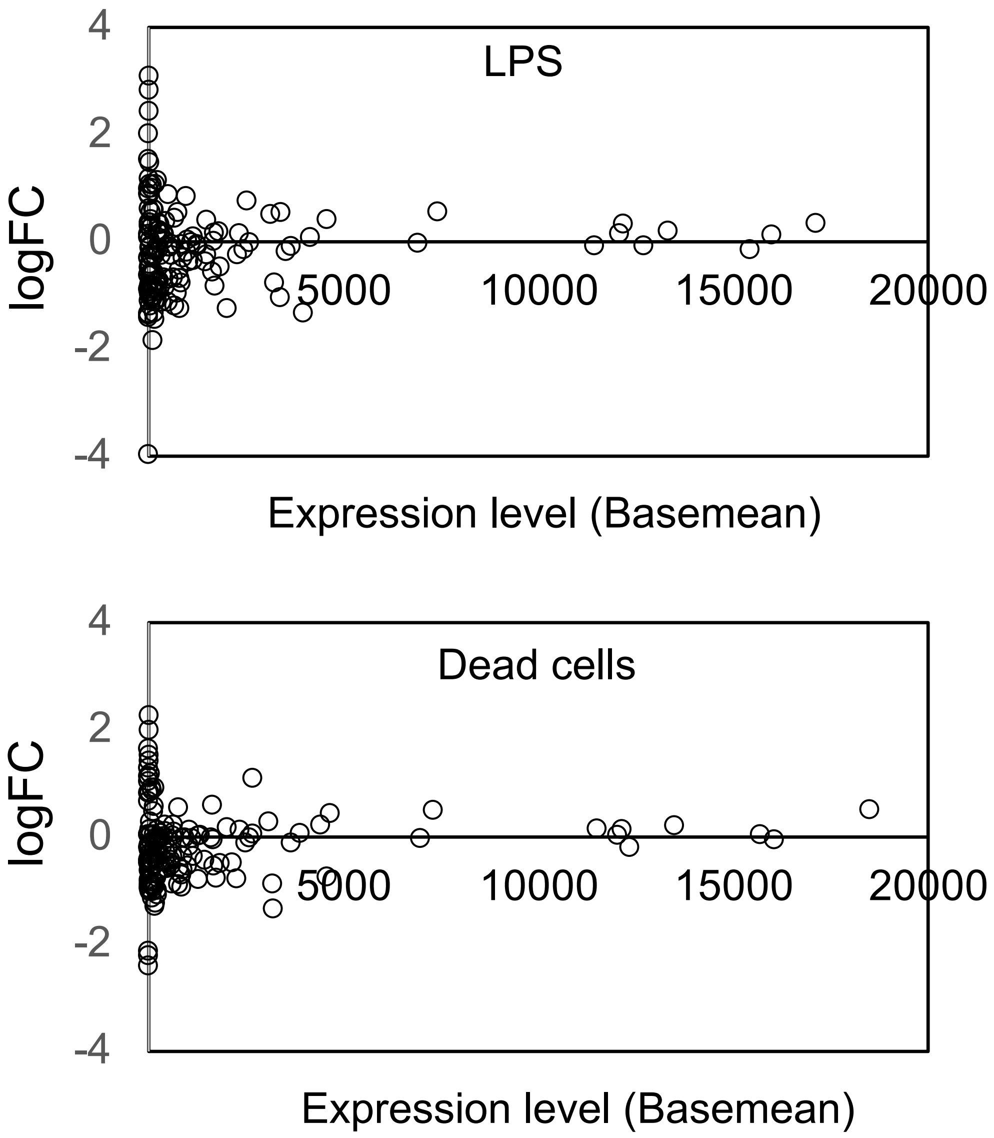

Figure S6 Plot of the logFC values and expression level (baseMean) of 149 peroxidase (ascorbate peroxidase and guaiacol peroxidase) genes.
