# Supplementary material for: Effects of LPS from Rhodobacter sphaeroides, a Purple Non-Sulfur Bacterium (PNSB), on the Gene Expression of Rice Root
Source: Microorganisms. 2023 Jun 28;11(7):1676. doi: 10.3390/microorganisms11071676 (PMC10383378; doi:10.3390/microorganisms11071676)
Supplement: Supplementary file 1 [file microorganisms-11-01676-s001.zip › Table S1.pdf]

Table S1: List of up-regulated (361 genes) and down-regulated (93 genes) in Figure 2 which showed Padj < 0.05 values in LPS or dead cells

		LPS				Dead cells		
GeneID	baseMean	logFC	padj		baseMean	logFC	padj	Description
Os06g0509900	145.7244	10.66506	0.224223		134.6966	10.54096	8.05E-12	Similar to Thionin. (Os06t0509900-00)
Os06g0514100	540.935	6.167816	0.000708		1697.784	7.817175	3.08E-58	Thionin, Antimicrobial peptide, Defence against root pathogens (Os06t0514100-01)
Os06g0513050	245.2583	6.14879	0.022096		504.7763	7.190397	5.42E-05	Conserved hypothetical protein. (Os06t0513050-01)
Os06g0514800	20.18003	6.839652	0.360485		20.41284	6.845489	0.008779	Conserved hypothetical protein. (Os06t0514800-00)
Os07g0598000	3.231603	4.141562	0.601321		9.072041	5.662006	0.027534	Similar to NADPH HC toxin reductase (Fragment). (Os07t0598000-01);Similar to NADPH HC toxin reductase (Fragment). (Os07t0598000-02)
Os10g0177300	20.56308	5.378024	0.006105		19.48063	5.280179	0.000234	Similar to Chalcone and stilbene synthases, N-terminal domain containing protein. (Os10t0177300-00)
Os04g0301500	35.6641	8.635412	1.38E-08		2.722173	4.910923	0.733147	Basic helix-loop-helix transcription activator, Control of disease resistance, Regulation of salicylic acid (SA) and jasmonic acid (JA) signaling (Os04t0301500-01)
Os11g0529036	5.914962	4.123683	0.400558		9.592993	4.830888	0.032533	Similar to Chalcone and stilbene synthases, N-terminal domain containing protein. (Os11t0529036-00)
Os03g0775000	6.90568	6.267757	0.004244		2.021591	4.482249	0.99986	Leucine carboxyl methyltransferase domain containing protein. (Os03t0775000-00)
Os02g0660800	60.85066	4.260407	0.00217		69.33689	4.454243	3.76E-06	Similar to OSIGBa0143N19.7 protein. (Os02t0660800-00)
Os11g0529150	11.64419	3.489763	0.449995		21.26108	4.388404	0.000831	Hypothetical gene. (Os11t0529150-00)
Os08g0391300	7.937672	0.630756	0.999758		66.00371	4.317732	0.034023	Conserved hypothetical protein. (Os08t0391300-01)
Os11g0603000	34.34896	4.335305	0.009762		30.93995	4.167615	1.15E-05	Basic helix-loop-helix dimerisation region bHLH domain containing protein. (Os11t0603000-01)
Os09g0555100	15.68343	1.913631	0.350689		58.61428	4.059376	0.009341	Similar to auxin Efflux Carrier family protein. (Os09t0555100-00)
Os03g0183500	262.0588	4.540899	1.12E-17		170.742	3.877031	3.24E-18	Protein of unknown function DUF581 family protein. (Os03t0183500-01)
Os04g0395800	20.43898	5.360582	1.80E-05		6.817476	3.704647	0.390874	Tify domain containing protein. (Os04t0395800-01)

Os11g0529100	11.7402	3.229448	0.484836		15.45315	3.649044	0.009083	Similar to Chalcone and stilbene synthases, N-terminal domain containing protein. (Os11t0529100-00)
Os09g0564700	9.991928	2.350171	0.333665		21.54557	3.584177	0.026551	Similar to Vignain. (Os09t0564700-00)
Os03g0142600	165.3476	3.103949	3.48E-08		216.6871	3.520073	3.53E-07	Myb transcription factor domain containing protein. (Os03t0142600-00)
Os07g0153000	12.12568	3.511223	0.027326		12.04041	3.484357	0.036289	A member of the JASMONATE ZIM-domain (JAZ) family (Os07t0153000-01)
Os01g0225500	67.66576	2.846403	0.03962		100.5874	3.470761	0.000155	Similar to 3-methyl-2-oxobutanoate hydroxymethyltransferase. (Os01t0225500-00)
Os12g0637400	8.856377	5.636611	0.007238		2.089709	3.444348	0.99986	Similar to Purple acid phosphatase (EC 3.1.3.2) (Fragment). (Os12t0637400-01)
Os10g0167900	142.3608	3.308983	0.369027		141.2312	3.285438	7.17E-05	Type III polyketide synthase (Os10t0167900-01)
Os10g0168000	109.2667	3.23644	0.461468		113.2672	3.283287	0.001065	Hypothetical protein. (Os10t0168000-00)
Os04g0639000	236.7125	2.771907	3.41E-05		313.1046	3.212986	4.01E-08	Conserved hypothetical protein. (Os04t0639000-01)
Os10g0491400	5.260096	1.827946	0.78023		11.87423	3.208487	0.030932	Conserved hypothetical protein. (Os10t0491400-01)
Os10g0168200	20.11112	2.655962	0.244387		27.29048	3.144711	0.000234	Hypothetical protein. (Os10t0168200-00)
Os06g0513781	164.5254	3.41285	0.002934		135.2173	3.088939	0.000536	Conserved hypothetical protein. (Os06t0513781-01)
Os07g0120000	73.49564	2.56785	0.065419		92.82308	2.945418	2.13E-11	Similar to predicted protein. (Os07t0120000-00)
Os10g0118200	28.11392	3.082736	0.005669		26.01849	2.944923	0.040491	Conserved hypothetical protein. (Os10t0118200-01)
Os12g0168150	6.295954	6.136194	0.015263		0.683318	2.9155	0.99986	Hypothetical gene. (Os12t0168150-00)
Os10g0162842	66.98586	1.538506	0.260163		145.618	2.89232	0.003971	Hypothetical protein. (Os10t0162842-00)
Os03g0289800	7.163347	5.324345	0.027326		1.438005	2.84957	0.99986	Similar to Leucoanthocyanidin dioxygenase-like protein. (Os03t0289800-01)
Os12g0168100	7.819754	6.44827	0.004223		0.648174	2.846688	0.99986	Similar to AP2 domain containing protein RAP2.6 (Fragment). (Os12t0168100-01)
Os10g0168300	21.23668	2.373493	0.346504		28.21588	2.843561	0.001305	Similar to Chalcone and stilbene synthases, N-terminal domain containing protein. (Os10t0168300-00)
Os05g0212750	24.44717	2.444948	0.460604		30.53797	2.814692	0.002602	Hypothetical protein. (Os05t0212750-00)
Os09g0341600	13.67648	2.880231	0.088865		13.05354	2.793653	0.028628	Cyclin-like F-box domain containing protein. (Os09t0341600-01)
Os03g0181100	291.8159	2.923655	2.95E-09		268.5558	2.775337	1.14E-09	Tify domain containing protein. (Os03t0181100-01);Tify domain containing protein. (Os03t0181100-02)

Os10g0162844	28.07211	1.851915	0.114678		47.67225	2.758703	0.012208	Wall-associated kinase domain containing protein. (Os10t0162844-00)
Os05g0161500	137.5747	2.731663	0.000359		140.2078	2.750859	2.90E-05	Calcium-activated (p)ppGpp synthetase, Ca²⁺-activated RelA-SpoT homolog 2 (Os05t0161500-01)
Os01g0946600	138.1034	3.645301	2.38E-08		79.21109	2.744144	7.62E-06	Conserved hypothetical protein. (Os01t0946600-01)
Os10g0391400	32.8428	1.871914	0.246463		53.39051	2.702588	0.008779	Jasmonate ZIM-domain (JAZ) protein, TIFY family protein, Negative regulation of JA signal transduction pathway, Activation of hypersensitive cell death (Os10t0391400-01)
Os03g0741100	163.404	3.289899	2.13E-11		112.4932	2.667015	3.76E-06	Basic helix-loop-helix transcription factor, Drought tolerance (Os03t0741100-01)
Os10g0118100	13.56098	2.85507	0.037338		11.99782	2.639255	0.514567	Conserved hypothetical protein. (Os10t0118100-01)
Os06g0109200	11.11813	1.477569	0.597481		20.55069	2.576812	0.02197	Protein of unknown function DUF6, transmembrane domain containing protein. (Os06t0109200-01)
Os02g0137700	106.938	4.249741	5.47E-20		36.42171	2.526501	8.27E-05	NAD(P)-binding domain containing protein. (Os02t0137700-01)
Os03g0676400	18.48885	4.793311	0.000597		4.295592	2.509053	0.99986	VQ domain containing protein. (Os03t0676400-01)
Os03g0860100	34.4903	5.358573	4.34E-06		5.402358	2.461769	0.99986	Ethylene response factor, Transcriptional activator, Positive regulation of disease resistance (Os03t0860100-01)
Os12g0478400	30.34482	2.903697	0.000493		23.49573	2.461725	0.01565	EGF-type aspartate/asparagine hydroxylation site domain containing protein. (Os12t0478400-00)
Os10g0392900	20.5331	2.41382	0.137947		21.09932	2.447375	0.010813	Similar to esterase. (Os10t0392900-00)
Os05g0583000	12.01636	4.127675	0.003429		4.162439	2.413288	0.99986	Similar to WRKY8. (Os05t0583000-01)
Os12g0227500	2569.799	2.403028	0.282644		2600.735	2.411373	1.60E-06	Similar to Beta-glucosidase aggregating factor. (Os12t0227500-00)
Os11g0644800	29.51209	2.739957	0.002404		24.04353	2.387545	0.290911	Similar to Tyrosine/nicotianamine aminotransferases family protein, expressed. (Os11t0644800-00)
Os12g0227766	2180.561	2.401842	0.285221		2105.635	2.329435	2.90E-05	Hypothetical protein. (Os12t0227766-00)
Os12g0228650	18.65771	2.014523	0.140568		22.27868	2.305493	0.040984	Hypothetical protein. (Os12t0228650-00)
Os10g0137300	35.17102	3.301571	0.016986		19.24092	2.297433	0.524656	Similar to BLN1-1. (Os10t0137300-00)
Os07g0115800	24.46876	3.284894	0.007877		13.32652	2.273327	0.99986	Hypothetical conserved gene. (Os07t0115800-00)

Os06g0513943	1359.001	2.182834	0.404807		1401.433	2.224408	4.37E-05	Conserved hypothetical protein. (Os06t0513943-01)
Os03g0180900	331.6668	3.088268	9.08E-24		198.588	2.21663	1.03E-10	Jasmonate ZIM-domain containing protein, Transcriptional repressor of JA signaling, Regulation of phosphate starvation responses, Pi homeostasis (Os03t0180900-01)
Os10g0113100	1016.464	1.757625	0.692848		1306.677	2.200757	0.043516	Aldo/keto reductase family protein. (Os10t0113100-01);Similar to cDNA clone:J033143K18, full insert sequence. (Os10t0113100-02)
Os02g0807900	99.01305	2.688626	3.28E-05		75.02591	2.198776	0.003818	Conserved hypothetical protein. (Os02t0807900-01)
Os02g0511400	53.33668	1.798356	0.216643		65.17316	2.149353	0.023556	Glucose/ribitol dehydrogenase family protein. (Os02t0511400-01)
Os05g0213150	181.1879	1.814347	0.307055		219.3822	2.148826	0.028628	Hypothetical protein. (Os05t0213150-00)
Os09g0409100	2088.919	1.238138	0.137947		3396.05	2.142564	8.27E-05	Conserved hypothetical protein. (Os09t0409100-01)
Os09g0491238	1642.005	1.152621	0.148599		2758.923	2.126902	0.012181	Hypothetical protein. (Os09t0491238-00)
Os09g0491100	1689.547	1.135144	0.153026		2855.319	2.122433	0.011316	Similar to Beta-primeverosidase (EC 3.2.1.149). (Os09t0491100-01);Similar to Beta-glucosidase isozyme 2. (Os09t0491100-02);Similar to Beta-glucosidase 30. (Os09t0491100-03)
Os08g0190100	108.6669	2.376925	0.0434		94.34276	2.116151	9.78E-05	Germin-like protein 8-11, Disease resistance (Os08t0190100-01)
Os05g0212550	185.4298	1.771242	0.270877		222.3106	2.089353	4.10E-05	Hypothetical protein. (Os05t0212550-00)
Os11g0260100	73.07886	2.861702	0.000152		46.60124	2.084384	0.127805	SAM dependent carboxyl methyltransferase family protein. (Os11t0260100-00)
Os12g0503000	1154.661	2.080638	3.47E-11		1161.662	2.076525	0.001221	Allantoin transporter, Ureide metabolism, Nitrogen molecular sensor (Os12t0503000-01);Similar to Ureide permease 4. (Os12t0503000-03);Similar to Ureide permease 4. (Os12t0503000-04);Similar to Allantoin permease. (Os12t0503000-05)
Os05g0586600	36.47537	3.708553	2.95E-09		13.44275	2.057107	0.285098	Similar to SIGE (RNA polymerase sigma subunit E); DNA binding / DNA-directed RNA polymerase/ sigma factor/ transcription factor. (Os05t0586600-01);Potential plastid sigma factor of RNA polymerase (Os05t0586600-02)
Os05g0212900	351.6929	1.788267	0.184124		404.54	2.031264	9.78E-05	Similar to Chalcone synthase J (EC 2.3.1.74) (Naringenin-chalcone

								synthase J). (Os05t0212900-00)
Os04g0310100	121.0892	2.686539	0.00494		82.8673	2.020834	0.000731	Similar to H0211A12.12 protein. (Os04t0310100-01)
Os10g0392400	121.7733	3.55024	5.97E-16		48.48961	2.010127	0.000134	Tify domain containing protein. (Os10t0392400-01)
Os05g0213000	308.45	1.706874	0.224223		363.5878	1.997103	5.61E-05	Hypothetical protein. (Os05t0213000-00)
Os02g0808000	80.88029	2.567922	0.000193		58.31905	1.981603	0.012676	Hypothetical protein. (Os02t0808000-00)
Os07g0118250	119.5568	1.803967	0.246463		132.111	1.975074	0.000263	Protein of unknown function DUF538 family protein. (Os07t0118250-00)
Os01g0946700	1950.396	3.174279	1.11E-25		942.1311	1.927956	7.84E-09	Similar to Glucan endo-1,3-beta-glucosidase GV (EC 3.2.1.39) ((1->3)-beta-glucan endohydrolase GV) ((1->3)-beta-glucanase isoenzyme GV) (Beta-1,3-endoglucanase GV). (Os01t0946700-01)
Os01g0124700	154.9147	3.044807	5.93E-09		81.15194	1.927938	0.013469	Hypothetical protein. (Os01t0124700-01)
Os12g0138800	193.4471	1.589112	0.022373		232.0921	1.915854	7.74E-05	Six-bladed beta-propeller, TolB-like domain containing protein. (Os12t0138800-01)
Os04g0308500	777.3825	2.294857	0.008102		611.5267	1.852461	0.001221	Similar to H0211A12.12 protein. (Os04t0308500-01)
Os01g0124650	210.8575	2.909909	6.01E-10		114.4936	1.841667	0.003818	Hypothetical conserved gene. (Os01t0124650-02)
Os04g0308400	771.9194	2.158627	0.023882		649.5043	1.832985	0.000234	Similar to H0211A12.13 protein. (Os04t0308400-00)
Os01g0544325	40.54163	0.94328	0.687763		63.38156	1.819564	0.043168	Non-protein coding transcript. (Os01t0544325-00)
Os06g0220900	134.9014	1.977139	0.000239		124.5506	1.815281	0.001118	Conserved hypothetical protein. (Os06t0220900-00)
Os08g0200100	57.72844	1.99597	0.042362		51.68036	1.779964	0.057472	Ribonucleotide reductase-related domain containing protein. (Os08t0200100-01)
Os11g0684000	133.8181	3.246165	3.99E-13		56.33285	1.756915	0.003497	Myb transcription factor, Jasmonate-dependent defense response, Negative regulation of bacterial blight resistance (Os11t0684000-01)
Os07g0178700	14.24146	4.386229	0.001291		2.859814	1.753591	0.99986	Similar to Low molecular mass early light-inducible protein HV90, chloroplast precursor (ELIP). (Os07t0178700-01)
Os04g0179100	20.69156	2.913673	0.004499		10.6468	1.740754	0.99986	Similar to OSIGBa0109M01.2 protein. (Os04t0179100-01)
Os10g0530200	5378.922	1.411216	0.058112		6406.932	1.733029	9.69E-05	Similar to Glutathione S-transferase TSI-1 (EC 2.5.1.18) (Glutathione S-transferase 1). (Os10t0530200-01)
Os05g0142100	100.6576	2.104719	0.000314		82.76498	1.732356	0.110708	Conserved hypothetical protein. (Os05t0142100-01)
Os12g0548700	2665.511	1.348194	1.06E-07		3241.349	1.71253	0.028252	Similar to MPI. (Os12t0548700-01)

Os10g0185400	350.331	1.784001	0.006416		337.5935	1.703052	0.386439	Pectinesterase inhibitor domain containing protein. (Os10t0185400-00)
Os11g0644650	291.6423	1.375818	0.001068		343.209	1.677596	0.004567	Hypothetical protein. (Os11t0644650-01)
Os12g0548401	967.2015	1.579724	0.000345		1008.867	1.644882	0.030932	Similar to Proteinase inhibitor. (Os12t0548401-01)
Os04g0658200	15.00724	4.461199	0.000991		2.688215	1.620611	0.99986	Similar to OSIGBa0132E09-OSIGBa0108L24.3 protein. (Os04t0658200-01)
Os11g0482901	166.6197	3.006514	1.01E-07		75.51282	1.617415	0.143504	Hypothetical gene. (Os11t0482901-01)
Os05g0192100	274.0193	1.455036	0.017399		299.027	1.611734	0.193231	Similar to Stem 28 kDa glycoprotein. (Os05t0192100-01)
Os11g0644700	323.6633	1.29188	0.00494		382.2877	1.605161	0.005533	Plant disease resistance response protein family protein. (Os11t0644700-01)
Os04g0423400	62.74815	1.590147	0.004545		63.11072	1.587669	0.139634	Similar to OSIGBa0076I14.3 protein. (Os04t0423400-01);Similar to OSIGBa0076I14.3 protein. (Os04t0423400-02)
Os02g0626600	134.1023	2.276042	0.010634		92.35027	1.582588	0.227889	Similar to Phenylalanine ammonia-lyase. (Os02t0626600-00)
Os03g0198600	219.6709	1.708285	0.015125		205.3844	1.564263	0.028811	Homeodomain-leucine zipper transcription factor, Regulation of panicle exsertion (Os03t0198600-01)
Os02g0181300	105.5535	1.747526	0.001667		96.58813	1.5627	0.027534	WRKY transcription factor, Defense response (Os02t0181300-01)
Os01g0950000	215.2661	1.33919	0.176127		242.378	1.55905	0.046251	Similar to Glutathione S-transferase GST 28 (EC 2.5.1.18) (Fragment). (Os01t0950000-01)
Os02g0120100	74.82876	1.747322	0.006647		67.9993	1.550366	0.066661	Serine/threonine/tyrosine protein kinase, STY kinase, Regulation of ammonium uptake (Os02t0120100-01);Similar to ATP binding protein. (Os02t0120100-02)
Os05g0142400	135.5088	1.903952	0.000133		113.1379	1.548941	0.000304	Conserved hypothetical protein. (Os05t0142400-01)
Os08g0298700	472.2201	1.618733	0.000142		457.3471	1.540536	0.06119	Similar to male sterility protein 2. (Os08t0298700-01)
Os04g0310200	55.55781	2.469738	0.043413		33.30797	1.535121	0.528152	Similar to H0211A12.13 protein. (Os04t0310200-00)
Os07g0475900	89.13859	1.500337	0.047046		90.86116	1.520335	0.003524	ACT domain containing protein kinase (Os07t0475900-01)
Os09g0455900	224.3523	1.177459	0.00225		267.6223	1.51855	0.30091	Alpha/beta hydrolase fold-3 domain containing protein. (Os09t0455900-01)
Os09g0455750	225.2901	1.157628	0.003469		270.3388	1.511591	0.310273	Hypothetical protein. (Os09t0455750-00)
Os01g0108600	140.8411	1.761036	0.000994		124.5931	1.510845	0.005051	Basic helix-loop-helix dimerisation region bHLH domain containing protein.

								(Os01t0108600-01);Hypothetical conserved gene. (Os01t0108600-02)
Os07g0138200	56.72313	1.373196	0.016134		61.31265	1.510033	0.009341	NAC transcription factor, ABA-induced leaf senescence and tillering (Os07t0138200-01)
Os07g0633400	129.8988	0.90104	0.029556		175.6041	1.509947	7.17E-05	IQ calmodulin-binding region domain containing protein. (Os07t0633400-01)
Os01g0834900	580.74	2.668071	2.58E-18		304.8068	1.500377	2.43E-09	Hypothetical conserved gene. (Os01t0834900-01)
Os01g0676800	72.93838	1.464308	0.003001		74.21375	1.482005	0.001124	Conserved hypothetical protein. (Os01t0676800-01)
Os04g0690800	110.6094	1.461774	1.32E-05		112.4182	1.479365	0.106402	22-kDa Photosystem II protein, Photoprotection (Os04t0690800-01)
Os03g0399800	83.40023	2.149059	0.003513		58.37526	1.473456	0.857172	Mannose-binding lectin domain containing protein. (Os03t0399800-01)
Os09g0364800	43.11122	0.817815	0.395943		58.3359	1.441533	0.023656	Heavy metal transport/detoxification protein domain containing protein. (Os09t0364800-01)
Os07g0637700	284.4284	1.205589	0.037338		321.7928	1.438325	0.14426	Similar to Disease resistance-responsive family protein. (Os07t0637700-00)
Os01g0186000	82.80743	4.716221	3.42E-09		11.20982	1.436235	0.99986	Similar to WRKY transcription factor 10. (Os01t0186000-01)
Os06g0166500	121.9138	1.157894	0.151604		140.3424	1.42739	0.046552	Aux/IAA protein, Mediation of abiotic stress tolerance, Drought and salt tolerance (Os06t0166500-01)
Os07g0633450	78.66433	0.805189	0.292118		106.391	1.42587	0.006995	Hypothetical gene. (Os07t0633450-00)
Os02g0654700	65.92097	2.687944	1.17E-05		32.55789	1.402385	0.506732	AP2/ERF family protein, Abiotic stress response (Os02t0654700-01)
Os01g0225600	58.51568	1.851216	0.036405		46.23088	1.387299	0.605994	Atypical 5C LEA (late embryogenesis abundant) protein, Multiple stress tolerance, Hg accumulation (Os01t0225600-01)
Os10g0133600	51.08793	1.227634	0.060382		55.53164	1.378618	0.013079	Protein kinase-like domain containing protein. (Os10t0133600-01)
Os01g0735500	34.8462	2.037651	0.014824		24.83668	1.377958	0.680029	Conserved hypothetical protein. (Os01t0735500-01)
Os02g0612900	59.27481	2.05509	4.27E-05		41.44331	1.367909	0.657076	Similar to Temperature stress-induced lipocalin. (Os02t0612900-01)
Os01g0225550	60.26673	1.83611	0.027392		47.40293	1.362392	0.577112	Hypothetical gene. (Os01t0225550-01)
Os10g0162856	419.6071	1.351509	0.337794		423.2235	1.354421	0.01565	Similar to Chalcone and stilbene synthases, N-terminal domain containing protein. (Os10t0162856-00)
Os05g0586200	5241.215	2.528499	6.41E-23		2774.058	1.35187	2.88E-06	Gretchen hagen 3 (GH3) family protein, Phytochrome, Jasmonyl-L-isoleucine synthase,

								Jasmonate signaling, Wound response, Response to blast infection (Os05t0586200-01)
Os07g0142500	1157.284	0.601583	0.774524		1630.187	1.331203	0.018934	Conserved hypothetical protein. (Os07t0142500-00)
Os01g0370000	1007.306	1.139927	0.048896		1113.616	1.330914	0.000765	NADH:flavin oxidoreductase/NADH oxidase, N-terminal domain containing protein. (Os01t0370000-01)
Os10g0530800	2727.837	1.35023	6.54E-05		2712.204	1.323445	0.550198	Hypothetical protein. (Os10t0530800-01)
Os08g0269700	202.1764	0.990668	0.303923		238.7445	1.322523	0.026551	Conserved hypothetical protein. (Os08t0269700-01)
Os12g0548501	1221.918	1.521365	5.93E-09		1111.879	1.318175	0.012181	Similar to CI2C. (Os12t0548501-01)
Os12g0267200	911.7223	1.457446	0.012883		857.409	1.317484	0.043168	Cyclopropane-fatty-acyl-phospholipid synthase domain containing protein. (Os12t0267200-01)
Os01g0735450	33.63243	1.976807	0.025256		24.05166	1.317315	0.707181	Hypothetical protein. (Os01t0735450-01)
Os04g0125700	4052.017	1.995805	3.02E-09		2856.215	1.31584	0.050176	Concanavalin A-like lectin/glucanase domain containing protein. (Os04t0125700-01)
Os10g0530900	2686.734	1.330548	9.50E-05		2670.392	1.302944	0.565095	Similar to Glutathione S-transferase GST 30 (EC 2.5.1.18). (Os10t0530900-01);Similar to cDNA clone:006-203-F02, full insert sequence. (Os10t0530900-03)
Os09g0248900	1092.46	1.040919	0.001239		1247.767	1.300606	0.010361	Myb/SANT-like domain domain containing protein. (Os09t0248900-01)
Os11g0483000	129.2512	2.771811	1.32E-05		57.49358	1.300403	0.772975	Cytochrome P450, Oxidase, JA-mediated chilling tolerance (Os11t0483000-01)
Os04g0541700	151.4412	0.96619	0.070804		177.8026	1.287759	0.017252	Similar to Homeobox-leucine zipper protein HOX22. (Os04t0541700-01);Homeodomain-leucine zipper (HD-Zip) protein, Transcription factor, ABA-mediated drought and salt tolerance (Os04t0541700-02)
Os11g0211800	386.2481	1.84628	0.030598		286.2187	1.249786	0.29644	Cysteine-rich peptide, Short-chain peptide, ABA-dependent drought tolerance (Os11t0211800-01)
Os05g0559316	569.8944	0.918748	0.000443		670.6357	1.247714	0.000263	Hypothetical conserved gene. (Os05t0559316-00)
Os01g0621900	1962.705	1.297754	6.91E-07		1929.733	1.245842	0.001221	Similar to OSIGBa0097P08.1 protein. (Os01t0621900-01);Similar to OSIGBa0097P08.1 protein. (Os01t0621900-02);Similar to OSIGBa0097P08.1 protein.

								(Os01t0621900-03);Hypothetical conserved gene. (Os01t0621900-04)
Os11g0141400	263.7348	1.117687	0.147048		282.1252	1.242501	0.042655	Similar to EF hand family protein, expressed. (Os11t0141400-01)
Os09g0439200	327.6254	1.9726	2.56E-17		225.7917	1.242014	9.78E-05	Jasmonate ZIM-domain protein, Jasmonate-induced resistance to bacterial blight, Repressor of jasmonic acid signaling (Os09t0439200-01)
Os01g0882100	42.1682	2.823702	2.65E-05		17.61086	1.235354	0.99986	Hypothetical conserved gene. (Os01t0882100-00)
Os01g0882000	72.17154	2.595911	1.53E-11		34.53042	1.229242	0.307071	Conserved hypothetical protein. (Os01t0882000-01)
Os03g0815100	742.4602	1.653558	0.025451		600.2905	1.220644	0.850898	NAC-domain transcription factor, Response to infection by M. grisea, MeJA-mediated signaling pathway, Drought and salinitytolerance (Os03t0815100-01)
Os10g0179000	33.11016	1.550593	0.032002		28.21753	1.212581	0.681659	Hypothetical protein. (Os10t0179000-00)
Os08g0502700	80.33446	2.620583	6.48E-06		37.31488	1.202513	0.991897	Pyridoxal phosphate-dependent transferase, major region, subdomain 1 domain containing protein. (Os08t0502700-01);Similar to serine--glyoxylate aminotransferase. (Os08t0502700-03)
Os08g0331900	51.0176	1.445682	0.011392		44.92469	1.172458	0.37864	Similar to Leucine Rich Repeat family protein, expressed. (Os08t0331900-01)
Os12g0131400	202.2295	1.541704	0.00108		168.8343	1.163482	0.193231	Hypothetical conserved gene. (Os12t0131400-01)
Os02g0204400	98.69614	2.250935	2.05E-10		55.63332	1.150763	0.358664	Similar to (6-4)DNA photolyase. (Os02t0204400-00)
Os01g0124401	1971.418	1.629143	0.010467		1553.586	1.138935	0.681866	Similar to Bowman-Birk type bran trypsin inhibitor. (Os01t0124401-01)
Os11g0521000	491.8229	0.455826	0.963275		668.6748	1.135779	0.043168	Lipase, GDSL domain containing protein. (Os11t0521000-01)
Os06g0231600	178.4934	1.529509	0.000158		147.7208	1.130514	0.011877	Similar to RING-H2 finger protein ATL1Q. (Os06t0231600-01)
Os06g0676400	139.6226	1.554623	0.000193		113.8858	1.12739	0.053763	Conserved hypothetical protein. (Os06t0676400-01)
Os09g0494600	225.2639	1.559855	0.000771		182.247	1.11889	0.99986	Protein of unknown function DUF599 family protein. (Os09t0494600-01)
Os11g0453900	17.49566	2.522598	0.016588		8.247856	1.115826	0.99986	Dehydrin RAB 16D. (Os11t0453900-01)
Os01g0314800	884.4457	1.315799	9.62E-09		808.0392	1.112824	0.021488	Late embryogenesis abundant protein 3 family protein. (Os01t0314800-01)
Os01g0124100	171.2448	2.315065	0.000238		91.10612	1.11069	0.99986	Proteinase inhibitor I12, Bowman-Birk family protein. (Os01t0124100-01)

Os08g0123833	86.8649	1.401437	0.033116		75.47129	1.099738	0.506732	Hypothetical protein. (Os08t0123833-00)
Os02g0753000	181.397	1.38956	0.000184		158.1441	1.090714	0.10327	Similar to Trehalose-6-phosphate phosphatase. (Os02t0753000-01)
Os03g0603600	428.1729	0.624043	0.657955		528.0139	1.076033	0.001221	PLC-like phosphodiesterase, TIM beta/alpha-barrel domain domain containing protein. (Os03t0603600-01)
Os10g0178800	116.8498	1.007915	0.031582		121.3929	1.070187	0.382144	Hypothetical conserved gene. (Os10t0178800-01)
Os08g0545700	48.68747	2.220467	6.37E-06		26.81834	1.062813	0.807863	Similar to TraB protein-related. (Os08t0545700-01)
Os12g0203200	3079.05	1.418943	4.33E-08		2604.446	1.057711	0.00174	Conserved hypothetical protein. (Os12t0203200-01)
Os03g0439650	221.733	0.167892	0.999758		321.3189	1.037183	0.022998	Hypothetical protein. (Os03t0439650-00)
Os04g0119500	1945.285	1.971254	1.00E-15		1215.101	1.034401	0.000223	Non-protein coding transcript. (Os04t0119500-01);Similar to Membrane related protein-like. (Os04t0119500-02);Similar to Membrane related protein-like. (Os04t0119500-03)
Os02g0643500	51.34919	2.084317	0.001527		29.92208	1.027223	0.99986	Pentapeptide repeat containing protein. (Os02t0643500-01)
Os06g0112100	3763.765	1.172922	1.11E-06		3540.491	1.026504	0.015771	Nucleoside phosphorylase, family 1 domain containing protein. (Os06t0112100-01)
Os06g0211200	310.0691	1.811606	0.000239		210.041	1.024118	0.705276	bZIP transcription factor, Positive regulator of ABA signaling, Drought tolerance (Os06t0211200-01);Similar to Abscisic acid responsive elements-binding factor (ABA-responsive element binding protein 1) (AREB1). (Os06t0211200-02)
Os05g0126800	722.0634	1.153567	0.000449		682.0588	1.015715	0.003818	Mss4-like domain containing protein. (Os05t0126800-01)
Os05g0183100	137.7012	1.965144	1.34E-06		84.96704	1.00054	0.255078	WRKY transcription factor, Positive regulation of blast and bacteria blight resistance (Os05t0183100-01)
Os06g0661800	87.17808	1.235791	0.005726		78.63607	0.99946	0.293757	Similar to Cryptochrome DASH, chloroplastic/mitochondrial. (Os06t0661800-01);Similar to Cryptochrome dash (Fragment). (Os06t0661800-02)
Os10g0542900	54.612	1.703479	0.037469		38.58645	0.990346	0.99986	Similar to chitinase. (Os10t0542900-01)
Os01g0609000	1166.829	0.739004	0.824453		1312.912	0.986047	0.026948	Similar to Pleiotropic drug resistance protein 3. (Os01t0609000-00)
Os01g0850900	1080.86	2.846264	1.27E-16		396.256	0.985763	0.146737	SOUL haem-binding protein domain containing

								protein. (Os01t0850900-01)
Os05g0127500	202.5405	2.077347	0.001363		116.1204	0.979172	0.99986	Similar to Leucoanthocyanidin dioxygenase-like protein. (Os05t0127500-01)
Os01g0812200	574.8128	0.548962	0.524124		695.749	0.968189	0.013174	Cyclin-like F-box domain containing protein. (Os01t0812200-01)
Os11g0204800	63.6126	1.247383	0.028744		56.15712	0.966776	0.337668	Similar to Wali7 protein (Fragment). (Os11t0204800-01)
Os06g0725250	44.05354	2.771401	3.41E-05		16.67558	0.957214	0.99986	Hypothetical protein. (Os06t0725250-00)
Os10g0105700	585.0974	0.984889	0.078847		577.6863	0.938248	0.009186	Similar to predicted protein. (Os10t0105700-01)
Os12g0227400	899.6997	0.48173	0.999758		1101.057	0.933368	0.000719	Allyl alcohol dehydrogenase. (Os12t0227400-01)
Os12g0227450	545.3734	0.442	0.999758		677.3953	0.93073	0.001221	Non-protein coding transcript. (Os12t0227450-00)
Os09g0442100	195.5158	1.348296	0.034272		160.9102	0.924201	0.565095	Protein kinase, core domain containing protein. (Os09t0442100-01)
Os05g0497300	745.6506	1.600032	0.000616		537.9441	0.917078	0.955813	Similar to Ethylene response factor 2. (Os05t0497300-01)
Os11g0129000	1491.347	1.623784	4.42E-08		1062.615	0.913352	0.126059	Similar to Transparent testa 12 protein. (Os11t0129000-00)
Os11g0129101	1293.189	1.609335	1.65E-07		928.3013	0.913079	0.167376	Non-protein coding transcript. (Os11t0129101-00)
Os01g0608900	508.8851	0.45794	0.999758		620.9017	0.90654	0.033382	Hypothetical gene. (Os01t0608900-01)
Os02g0683900	1342.877	1.27925	1.51E-05		1131.77	0.898168	0.02228	Protein of unknown function DUF1084 family protein. (Os02t0683900-01);Similar to T5E21.3. (Os02t0683900-02)
Os03g0402800	2106.674	1.712575	1.06E-11		1420.237	0.894661	0.038213	TIFY family protein, JASMONATE-ZIM domain (JAZ) protein, JA signaling, Regulation of spikelet development (Os03t0402800-01);Similar to ZIM motif family protein, expressed. (Os03t0402800-02)
Os01g0597600	18.52976	3.041464	0.042265		5.837059	0.892065	0.99986	Amino acid transporter, transmembrane domain containing protein. (Os01t0597600-01)
Os03g0176700	71.32945	2.032648	1.04E-05		40.1824	0.878626	0.99986	Conserved hypothetical protein. (Os03t0176700-00)
Os05g0149400	312.7602	3.008296	0.001745		98.4824	0.874146	0.99986	ACC oxidase, Ethylene biosynthesis (Os05t0149400-01)
Os05g0497200	80.53069	1.823125	0.047708		49.92598	0.845657	0.99986	AP2/ERF (APETALA2/ethylene-responsive factor) protein, Regulation of spikelet meristem determinancy and

								floral organ identity (Os05t0497200-01)
Os12g0626400	339.7449	0.815514	0.025451		346.0681	0.839243	0.030932	Similar to Phytoene synthase 1, chloroplast precursor (EC 2.5.1.-) (Fruit ripening specific protein pTOM5). (Os12t0626400-01);Phytoene synthase 2, Carotenoid biosynthesis (Os12t0626400-02)
Os03g0308800	127.0419	1.957101	4.39E-09		72.95058	0.832253	0.79843	Tetratricopeptide-like helical domain containing protein. (Os03t0308800-01);Hypothetical conserved gene. (Os03t0308800-02);Hypothetical conserved gene. (Os03t0308800-03)
Os05g0579500	58.20514	1.255896	0.035572		48.12696	0.830652	0.767474	Similar to 10A19I.7. (Os05t0579500-01)
Os01g0215500	166.815	2.363828	1.47E-06		75.92709	0.8293	0.99986	Similar to esterase. (Os01t0215500-01)
Os01g0371500	8306.163	1.005934	0.002475		7726.827	0.827527	0.05213	Similar to Glutathione-S-transferase 19E50. (Os01t0371500-01)
Os02g0572500	1677.341	1.163525	8.26E-05		1445.825	0.82291	0.130055	Non-protein coding transcript. (Os02t0572500-00)
Os03g0730433	678.091	1.302103	0.000336		545.2417	0.819382	0.814055	Hypothetical protein. (Os03t0730433-00)
Os03g0730400	690.2346	1.302293	0.000491		554.4681	0.817416	0.842789	Similar to Serine carboxypeptidase. (Os03t0730400-01);Similar to Serine carboxypeptidase family protein, expressed. (Os03t0730400-02)
Os06g0676300	164.3871	1.327597	0.003021		130.358	0.815672	0.535404	Non-protein coding transcript. (Os06t0676300-02)
Os02g0799000	945.0245	0.73241	0.085676		988.1056	0.814816	0.043168	Protein phosphatase 2C 27, Negative regulation of cold tolerance (Os02t0799000-01)
Os10g0530750	324.5443	0.951804	0.000448		306.2433	0.805003	0.108002	Hypothetical protein. (Os10t0530750-00)
Os03g0290300	710.6947	1.607514	1.61E-05		485.1962	0.800769	0.404109	Similar to W-3 fatty acid desaturase (Fragment). (Os03t0290300-01)
Os09g0464066	36.5933	2.027671	0.016115		19.92394	0.800423	0.99986	Hypothetical protein. (Os09t0464066-00)
Os01g0658900	542.8458	1.128594	0.018761		468.3966	0.788171	0.170285	bZIP class abscisic acid responsive element (ABRE)-binding factor, Salt tolerance (Os01t0658900-01)
Os11g0267300	652.0601	1.467845	6.31E-07		475.1768	0.783156	0.174397	Similar to Endonuclease III homologue. (Os11t0267300-01)
Os01g0687300	1530.743	1.193927	7.71E-05		1271.053	0.77101	0.169921	Conserved hypothetical protein. (Os01t0687300-01);Conserved hypothetical protein. (Os01t0687300-02)
Os05g0186900	74.05651	2.528519	0.000226		29.76325	0.768468	0.99986	Similar to Auxin-responsive protein IAA16.

								(Os05t0186900-01)
Os01g0246200	97.26005	1.961034	0.001996		54.06192	0.764131	0.99986	Similar to TCP-domain protein. (Os01t0246200-00)
Os06g0725200	58.29276	2.475678	3.41E-05		24.15888	0.763285	0.99986	Lipase, GDSL domain containing protein. (Os06t0725200-01)
Os04g0412100	47.76364	2.891808	1.14E-08		15.3829	0.761937	0.99986	Similar to H0717B12.8 protein. (Os04t0412100-01)
Os02g0572400	3379.44	1.195354	4.21E-05		2789.293	0.758936	0.282038	Similar to Riboflavin biosynthesis protein ribA, chloroplast precursor [Includes: GTP cyclohydrolase II (EC 3.5.4.25); 3,4-dihydroxy-2-butanone 4- phosphate synthase (DHBP synthase)]. (Os02t0572400-01);Similar to predicted protein. (Os02t0572400-02)
Os01g0705200	32.18241	2.863022	0.015434		10.55447	0.758916	0.99986	Late embryogenesis abundant protein repeat containing protein. (Os01t0705200-01)
Os05g0582300	870.6478	1.331489	7.76E-11		670.9907	0.755347	0.239933	Solanesyl diphosphate synthase, Providing solanesyl diphosphate for plastoquinone-9 (PQ-9) formation (Os05t0582300-01)
Os05g0582350	461.2915	1.318571	1.64E-07		357.771	0.754717	0.258722	Hypothetical protein. (Os05t0582350-00)
Os02g0600200	65.69185	1.664174	0.001952		42.59182	0.751376	0.99986	IQ calmodulin-binding region domain containing protein. (Os02t0600200-01);Similar to predicted protein. (Os02t0600200-02)
Os04g0564700	427.0836	0.985857	0.012291		386.6488	0.745296	0.483419	WD40 repeat domain containing protein. (Os04t0564700-01);Similar to OSIGBa0158F05.3 protein. (Os04t0564700-02)
Os03g0235400	134.5055	0.987971	0.024762		121.4404	0.744339	0.732748	Hypothetical protein. (Os03t0235400-01)
Os05g0127200	1663.173	1.028792	0.033116		1473.56	0.743193	0.99986	Similar to Phospholipase C (Fragment). (Os05t0127200-01);Similar to Phospholipase C (Fragment). (Os05t0127200-02)
Os12g0134000	661.4288	0.761129	0.007483		661.7177	0.74305	0.075611	Similar to Hydroxymethylglutaryl-CoA lyase. (Os12t0134000-01);Similar to hydroxymethylglutaryl-CoA lyase. (Os12t0134000-02)
Os05g0127266	628.9227	0.986013	0.017472		568.0853	0.740929	0.99986	Non-protein coding transcript. (Os05t0127266-00)
Os11g0694125	150.0213	1.293429	0.040654		116.9365	0.738226	0.99986	Hypothetical protein. (Os11t0694125-00)
Os07g0615200	6088.244	1.333785	9.08E-09		4646.14	0.735042	0.066661	Tify domain containing protein. (Os07t0615200-01)

Os05g0149450	269.8451	2.884153	0.005726		86.03796	0.72977	0.99986	Non-protein coding transcript. (Os05t0149450-00)
Os01g0775100	3255.057	1.193357	7.64E-05		2648.727	0.72382	0.326568	Plus-3 domain containing protein. (Os01t0775100-01)
Os01g0676900	1637.608	0.92632	0.001045		1509.202	0.722757	0.119372	Hypothetical conserved gene. (Os01t0676900-01)
Os02g0816600	165.4405	1.764658	1.19E-06		100.1244	0.714206	0.850898	Similar to ABC(yeast) homolog1. (Os02t0816600-01);Similar to ABC1 family protein. (Os02t0816600-02)
Os01g0124200	5080.464	1.321997	0.000241		3854.073	0.70807	0.99986	Bowman-Birk type trypsin inhibitor, Target of a fungal effector and an NLR receptor for innate immunity, Resistance to fungal pathogen M. oryzae (Os01t0124200-02)
Os02g0203000	432.9915	1.480074	5.89E-05		303.5258	0.707957	0.707181	bZIP transcription factor, Homolog of Arabidopsis HY5 transcription factor, Photomorphogenesis (Os02t0203000-01)
Os01g0246400	165.6733	2.107718	0.000419		82.31755	0.69431	0.99986	Similar to Low molecular mass early light-inducible protein HV90, chloroplast precursor (ELIP). (Os01t0246400-01)
Os05g0542200	3231.101	1.061906	4.95E-06		2754.23	0.687524	0.047213	Alpha/beta hydrolase fold-1 domain containing protein. (Os05t0542200-01);Similar to catalytic/ hydrolase. (Os05t0542200-02);Similar to cDNA clone:J023112L03, full insert sequence. (Os05t0542200-03)
Os03g0245100	641.4068	0.995528	0.012291		563.637	0.685265	0.638892	NAD(P)-binding domain containing protein. (Os03t0245100-01);Similar to Dihydrodipicolinate reductase. (Os03t0245100-02);NAD(P)-binding domain containing protein. (Os03t0245100-03)
Os11g0691300	479.412	1.11866	0.00225		397.1397	0.682274	0.290911	Similar to Protein kinase. (Os11t0691300-00)
Os05g0578750	1054.68	0.883362	0.001073		973.326	0.680933	0.192832	Hypothetical protein. (Os05t0578750-00)
Os05g0292800	57.83938	1.63914	0.041416		36.90271	0.680322	0.99986	Similar to One helix protein (OHP). (Os05t0292800-01)
Os05g0542150	1435.539	0.967027	0.000965		1274.411	0.679063	0.151852	Hypothetical gene. (Os05t0542150-01)
Os01g0775150	771.2827	1.046552	0.006106		658.6237	0.673672	0.640465	Non-protein coding transcript. (Os01t0775150-00)
Os05g0578600	1365.723	0.923476	0.000491		1233.653	0.673113	0.150903	Similar to Polygalacturonase PG2. (Os05t0578600-01)
Os11g0694100	209.7272	1.1788	0.043413		167.445	0.664451	0.99986	Similar to Protein kinase domain containing protein, expressed. (Os11t0694100-01)

Os11g0691100	19710.05	1.212693	6.36E-06		15358.47	0.644419	0.535404	Similar to Protein kinase. (Os11t0691100-00)
Os03g0390200	1334.462	1.270951	7.86E-11		1009.182	0.641266	0.075835	Serine/threonine protein kinase, Hyperosmotic stress response (Os03t0390200-01);Serine/threonine-protein kinase SAPK1 (EC 2.7.1.37) (Osmotic stress/abscisic acid-activated protein kinase 1). (Os03t0390200-02)
Os11g0691050	19070.17	1.198367	1.56E-05		14923.07	0.638038	0.606991	Hypothetical protein. (Os11t0691050-00)
Os08g0440100	1830.376	1.182633	0.003814		1439.211	0.633787	0.970711	Similar to Temperature stress-induced lipocalin. (Os08t0440100-01)
Os01g0880250	2344.621	0.722816	0.015785		2273.455	0.630789	0.364018	Hypothetical protein. (Os01t0880250-00)
Os01g0880200	2733.41	0.742922	0.009588		2620.216	0.624206	0.378434	Xylan glucuronosyltransferase, Accumulation of cellulose and hemicellulose in the cell wall deposition (Os01t0880200-01);Similar to secondary cell wall-related glycosyltransferase family 8. (Os01t0880200-02);Similar to cDNA clone:J013112C08, full insert sequence. (Os01t0880200-03)
Os08g0501500	116.5003	1.277113	0.00261		87.14702	0.623404	0.99986	EGF-type aspartate/asparagine hydroxylation site domain containing protein. (Os08t0501500-00)
Os03g0781750	923.0547	0.928541	0.000117		813.9838	0.622173	0.303964	Non-protein coding transcript. (Os03t0781750-00)
Os06g0215900	3964.686	1.366348	1.77E-05		2832.583	0.619915	0.99986	Similar to Oxo-phytodienoic acid reductase. (Os06t0215900-01);Similar to 12-oxophytodienoic acid reductase. (Os06t0215900-02)
Os06g0669400	2078.758	1.08502	0.000202		1702.075	0.617668	0.143504	ATP-dependent zinc metalloprotease, Chloroplast development (Os06t0669400-01);Similar to Cell division protease ftsH homolog 2, chloroplastic. (Os06t0669400-02);Similar to ATP-dependent zinc metalloprotease FTSH 2, chloroplastic. (Os06t0669400-03)
Os04g0517100	1658.577	0.739578	0.035539		1585.334	0.614472	0.021728	Similar to OSIGBa0145M07.4 protein. (Os04t0517100-01);SG2-type MYB transcription factor, Cold tolerance, Resistance to fungal and bacterial

								pathogens, Panicle development (Os04t0517100-02)
Os06g0215925	4046.206	1.36691	1.77E-05		2880.31	0.611831	0.99986	Non-protein coding transcript. (Os06t0215925-00)
Os03g0781700	1263.335	0.954648	1.48E-05		1095.711	0.611062	0.29642	Reticulon family protein. (Os03t0781700-01)
Os04g0623300	3998.37	1.241803	1.17E-05		3024.317	0.607408	0.529143	Polyamine oxidase, Seed germination (Os04t0623300-01);Similar to H0215F08.3 protein. (Os04t0623300-02);Similar to H0215F08.3 protein. (Os04t0623300-03);Similar to H0215F08.3 protein. (Os04t0623300-04)
Os11g0134300	1779.625	0.816728	0.041416		1639.255	0.605816	0.849414	Similar to Serine/threonine kinase. (Os11t0134300-01)
Os02g0222100	949.0165	1.026071	0.028455		793.2598	0.600357	0.881556	Similar to Cysteine synthase (EC 4.2.99.8). (Os02t0222100-01)
Os03g0767500	348.5397	0.865335	0.003001		312.6743	0.595629	0.241688	Thioredoxin domain 2 containing protein. (Os03t0767500-01)
Os02g0711400	459.3779	0.993237	0.003021		387.6616	0.588629	0.750034	Conserved hypothetical protein. (Os02t0711400-01)
Os09g0401300	2417.472	1.452998	2.13E-19		1618.989	0.568303	0.040984	A member of the JASMONATE ZIM-domain (JAZ) family, Negative modulation of JA signaling and antiviral defense (Os09t0401300-01);Similar to ZIM motif family protein. (Os09t0401300-02);Similar to ZIM motif family protein. (Os09t0401300-03)
Os05g0320800	891.6616	0.919278	0.002418		769.7619	0.561873	0.640465	Hypothetical protein. (Os05t0320800-01)
Os01g0362100	608.6687	0.808728	0.007986		546.3794	0.537054	0.675826	Esterase/lipase/thioesterase domain containing protein. (Os01t0362100-01)
Os08g0288000	1146.773	0.727467	0.000719		1065.058	0.53243	0.174397	Conserved hypothetical protein. (Os08t0288000-01);Conserved hypothetical protein. (Os08t0288000-02)
Os01g0797600	870.8832	1.261179	3.02E-09		630.5082	0.525366	0.99986	AP2/ERF family protein, ERF-associated EAR-motif-containing repressor, Abiotic stress response, Stress signaling (Os01t0797600-01)
Os06g0590301	619.6777	1.157045	1.34E-05		471.5598	0.524045	0.817442	Similar to short-chain dehydrogenase/reductase (SDR) family protein. (Os06t0590301-01)
Os07g0123800	142.0341	1.351771	0.046918		97.96764	0.521246	0.99986	Uncharacterised conserved protein UCP031279 domain containing protein. (Os07t0123800-01)
Os09g0554000	90.60691	1.300265	0.003469		64.07874	0.511237	0.99986	Similar to Mitochondrial phosphate transporter. (Os09t0554000-01)

Os04g0583101	142.7454	1.427797	1.36E-05		94.50208	0.509842	0.99986	Pentatricopeptide repeat domain containing protein. (Os04t0583101-01)
Os04g0619000	216.8178	1.359869	0.02753		147.7975	0.500192	0.99986	Similar to L1332.9 protein. (Os04t0619000-01)
Os11g0216300	625.5124	0.75841	0.024272		565.6317	0.49962	0.535404	ABC-1 domain containing protein. (Os11t0216300-01);Similar to ABC1 family protein, expressed. (Os11t0216300-02)
Os01g0847200	2393.879	0.706446	0.015568		2213.415	0.498766	0.99986	Similar to Mannose-1-phosphate guanyltransferase (EC 2.7.7.13) (ATP-mannose-1-phosphate guanylyltransferase) (GDP-mannose pyrophosphorylase) (NDP-hexose pyrophosphorylase). (Os01t0847200-01);Similar to Mannose-1-phosphate guanyltransferase (EC 2.7.7.13) (ATP-mannose-1-phosphate guanylyltransferase) (GDP-mannose pyrophosphorylase) (NDP-hexose pyrophosphorylase). (Os01t0847200-02)
Os01g0739200	834.7512	0.896682	0.000223		707.3245	0.490591	0.884022	Protein-tyrosine phosphatase, dual specificity domain containing protein. (Os01t0739200-01)
Os06g0215750	8232.544	1.428927	2.37E-05		5397.484	0.487986	0.99986	Hypothetical gene. (Os06t0215750-00)
Os01g0847250	2053.254	0.701811	0.018554		1893.864	0.487856	0.99986	Non-protein coding transcript. (Os01t0847250-00)
Os06g0215600	8064.925	1.416628	2.37E-05		5302.384	0.479464	0.99986	Similar to Oxo-phytodienoic acid reductase. (Os06t0215600-02)
Os02g0620400	152.4294	1.292305	0.040906		105.6914	0.455945	0.99986	RmlC-like jelly roll fold domain containing protein. (Os02t0620400-01)
Os12g0263000	6910.11	0.52998	0.031184		6741.443	0.449115	0.655575	Similar to Glutathione synthase. (Os12t0263000-01);Hydroxymethyl-glutathione synthetase (Os12t0263000-02)
Os03g0252100	316.974	1.424252	0.005955		204.455	0.443642	0.99986	Alpha/beta hydrolase fold-3 domain containing protein. (Os03t0252100-01)
Os03g0273800	17278.65	0.843302	0.000116		14648.29	0.432524	0.787682	HAD-superfamily hydrolase, subfamily IA, variant 3 domain containing protein. (Os03t0273800-01)
Os04g0558400	706.7844	0.803429	0.00494		609.6334	0.431312	0.698939	RmlC-like jelly roll fold domain containing protein. (Os04t0558400-01);Similar to acyl-CoA thioesterase family protein. (Os04t0558400-02)

Os09g0362500	788.3718	1.115784	1.35E-09		588.7336	0.429881	0.99986	Peptidase M1, membrane alanine aminopeptidase family protein. (Os09t0362500-01)
Os10g0567400	396.7085	1.799577	4.35E-05		207.9474	0.411536	0.99986	Chlorophyllide a oxygenase, Chlorophyll b synthesis, Regulation of photosynthesis, Chlorophyll degradation and ROS scavenging, Regulation of both natural and induced senescence (Os10t0567400-01);Similar to Isoform 2 of Chlorophyllide a oxygenase, chloroplastic. (Os10t0567400-02);Similar to Isoform 2 of Chlorophyllide a oxygenase, chloroplastic. (Os10t0567400-03)
Os05g0136200	1269.473	0.984164	0.000994		1000.254	0.408064	0.99986	Serine/threonine protein kinase domain containing protein. (Os05t0136200-01);Serine/threonine protein kinase domain containing protein. (Os05t0136200-02)
Os08g0104900	734.1032	0.64511	0.015368		671.6902	0.4075	0.699343	Protein of unknown function DUF6, transmembrane domain containing protein. (Os08t0104900-01);Protein of unknown function DUF6, transmembrane domain containing protein. (Os08t0104900-02)
Os05g0320300	442.4823	0.872933	0.001678		366.2484	0.406029	0.99986	Similar to Homeobox protein. (Os05t0320300-01)
Os09g0464000	1636.932	0.74657	0.012142		1432.174	0.40508	0.99986	Similar to Carbonate dehydratase-like protein. (Os09t0464000-01);Similar to Carbonate dehydratase-like protein. (Os09t0464000-02)
Os04g0583000	157.2013	1.305542	2.09E-05		106.0187	0.404717	0.99986	Conserved hypothetical protein. (Os04t0583000-01)
Os09g0464033	1655.986	0.739124	0.01248		1450.457	0.399716	0.99986	Hypothetical gene. (Os09t0464033-01)
Os02g0254700	1800.028	0.491732	0.043171		1745.204	0.394302	0.99986	Similar to Spermidine synthase 3. (Os02t0254700-01)
Os01g0804400	81.48327	1.212375	0.013908		56.90357	0.379491	0.99986	Cytochrome P450 family protein. (Os01t0804400-01)
Os01g0159300	1080.7	0.714989	0.0488		948.8192	0.378226	0.99986	Zinc finger, RING/FYVE/PHD-type domain containing protein. (Os01t0159300-01)
Os01g0305200	484.4717	0.981237	0.000226		376.8695	0.37136	0.99986	Lg106-like family protein. (Os01t0305200-01)
Os08g0243500	3342.156	0.677822	0.000415		2971.263	0.370331	0.79843	Similar to NADPH-cytochrome P450 oxydoreductase isoform 2. (Os08t0243500-01);Similar to NADPH--cytochrome P450 reductase. (Os08t0243500-02)

Os01g0615100	279.1895	2.53456	7.87E-06		94.79338	0.362392	0.99986	Chymotrypsin protease inhibitor, Salt and osmotic stress tolerance (Os01t0615100-01)
Os04g0600200	584.5172	1.382212	0.005955		372.7381	0.360394	0.99986	Homodimeric diiron-carboxylate protein, Cyanide-resistant respiration in mitochondria, Reactive oxygen species (ROS) scavenging (Os04t0600200-01)
Os02g0179225	217.9436	1.217672	0.000493		150.5056	0.357577	0.99986	Hypothetical protein. (Os02t0179225-00)
Os07g0672566	155.7467	1.253403	0.001309		105.906	0.357331	0.99986	Hypothetical gene. (Os07t0672566-00)
Os04g0105100	348.9116	1.009256	0.027241		265.8961	0.352274	0.99986	Zinc finger, RING/FYVE/PHD-type domain containing protein. (Os04t0105100-01);RING E3 ligase, Negative regulation of salt stress response (Os04t0105100-02)
Os09g0544300	1067.189	0.638538	0.00201		957.7734	0.351964	0.99986	ATMRK serine/threonine protein kinase-like domain containing protein. (Os09t0544300-01)
Os10g0392500	230.6719	0.964663	0.022071		178.9702	0.346515	0.99986	Similar to RNA methyltransferase, TrmH family protein, expressed. (Os10t0392500-00)
Os05g0179100	398.8255	-0.710649	0.027596		566.3251	0.344015	0.99986	DVL family protein. (Os05t0179100-01)
Os06g0318566	129.4327	1.604804	0.000131		73.15277	0.340924	0.99986	Hypothetical protein. (Os06t0318566-00)
Os02g0179200	245.7202	1.193679	0.000672		170.2973	0.336882	0.99986	Glutamine amidotransferase class-I domain containing protein. (Os02t0179200-01)
Os03g0156700	1127.221	0.739678	0.001562		961.1798	0.331011	0.99986	Zebra-necrosis like protein, Nickel/cobalt transporter, high-affinity domain containing protein (Os03t0156700-01)
Os06g0634300	796.51	0.714619	0.005327		685.2567	0.326475	0.99986	Similar to cDNA clone:J023074C15, full insert sequence. (Os06t0634300-01);Similar to Nudix hydrolase 2 (EC 3.6.1.-) (AtNUDT2) (ADP-ribose pyrophosphatase) (EC 3.6.1.13) (NADH pyrophosphatase) (EC 3.6.1.22). (Os06t0634300-02)
Os04g0653000	6158.263	0.669641	0.013931		5386.171	0.318287	0.99986	Jasmonic acid (JA) signalling repressor, Regulation of spikelet development (Os04t0653000-01)
Os06g0154400	253.756	0.97834	0.001988		193.5151	0.317402	0.99986	Domain of unknown function DUF676, lipase-like domain containing protein. (Os06t0154400-01)

Os06g0318600	132.1952	1.560999	0.000239		75.66229	0.314568	0.99986	Peptidase S41 domain containing protein. (Os06t0318600-01)
Os01g0168100	1664.117	0.651931	0.046294		1463.878	0.313662	0.99986	KIP1-like domain containing protein. (Os01t0168100-01);Similar to CENP-E like kinetochore protein. (Os01t0168100-02)
Os06g0574100	290.9903	1.043864	0.000783		214.3476	0.30545	0.99986	Similar to predicted protein. (Os06t0574100-00)
Os11g0186200	9594.73	0.638157	0.028279		8452.035	0.302706	0.99986	Similar to Aldehyde dehydrogenase (EC 1.2.1.3). (Os11t0186200-01)
Os12g0617800	413.202	1.580236	6.03E-06		233.0062	0.299687	0.99986	Cation/H+ exchanger domain containing protein. (Os12t0617800-01)
Os07g0672600	188.9581	1.242752	0.002323		126.1376	0.296057	0.99986	Hypothetical conserved gene. (Os07t0672600-00)
Os01g0753100	709.1655	0.785325	0.000226		584.4158	0.294732	0.99986	Alcohol dehydrogenase superfamily, zinc-containing protein. (Os01t0753100-01);Similar to reticulon-4-interacting protein 1. (Os01t0753100-02)
Os03g0259100	2568.062	-0.911238	0.016409		3752.322	0.286529	0.99986	Similar to Basic blue protein (Cusacyanin) (Plantacyanin) (CBP). (Os03t0259100-01)
Os05g0566450	2514.233	0.718293	0.016588		2125.221	0.283768	0.99986	Non-protein coding transcript. (Os05t0566450-00)
Os02g0165100	688.6634	0.682792	0.029931		590.5033	0.281439	0.99986	Protein kinase, core domain containing protein. (Os02t0165100-01)
Os04g0461600	48.9972	1.305	0.032319		31.40059	0.271289	0.99986	Similar to Fw2.2. (Os04t0461600-01)
Os05g0144100	729.6811	0.855077	0.02363		578.0259	0.271145	0.99986	WD40 repeat-like domain containing protein. (Os05t0144100-01)
Os05g0569300	899.1932	1.205558	6.54E-05		602.2806	0.258098	0.99986	Similar to G-box binding factor 1. (Os05t0569300-01)
Os01g0534900	9194.719	0.539748	0.039895		8266.406	0.248204	0.99986	Similar to Hv711N16.16 (Fragment). (Os01t0534900-01)
Os05g0114400	227.2454	-1.239005	0.046245		351.2739	0.245065	0.99986	Zinc finger, C2H2-type domain containing protein. (Os05t0114400-01)
Os07g0150100	5054.455	0.64766	0.001555		4332.343	0.240964	0.99986	Protein of unknown function DUF221 domain containing protein. (Os07t0150100-01)
Os05g0257100	240.8368	0.969795	0.034332		178.2011	0.227852	0.99986	Leucine-rich repeat, plant specific containing protein. (Os05t0257100-01);Similar to SHR5-receptor-like kinase (Fragment). (Os05t0257100-02)
Os08g0136001	1225.337	0.745155	0.006113		1000.208	0.222235	0.99986	Hypothetical gene. (Os08t0136001-00)
Os01g0717000	2730.697	0.835506	0.004247		2137.959	0.2168	0.99986	Similar to GmCK1p (EC 2.7.1.32). (Os01t0717000-

								01);Similar to Choline kinase. (Os01t0717000-02);Similar to Choline kinase. (Os01t0717000-05)
Os03g0654400	834.5036	1.097705	3.39E-08		576.9926	0.206046	0.99986	Similar to Cationic amino acid transporter-like protein. (Os03t0654400-01);Similar to High-affinity cationic amino acid transporter 1. (Os03t0654400-02)
Os01g0535001	5217.268	0.5817	0.022329		4530.268	0.201765	0.99986	Non-protein coding transcript. (Os01t0535001-00)
Os04g0671100	513.8027	0.846702	0.001068		396.9409	0.194283	0.99986	Similar to H0624F09.8 protein. (Os04t0671100-01)
Os07g0125500	1387.365	0.700837	0.017826		1141.999	0.193439	0.99986	Allergen V5/Tpx-1 related family protein. (Os07t0125500-01)
Os01g0127600	187.8271	1.636858	0.001505		98.42601	0.19026	0.99986	Similar to Bowman-Birk type proteinase inhibitor D-II precursor (IV). (Os01t0127600-01)
Os01g0757301	110.7124	1.806684	0.000448		53.10051	0.188476	0.99986	Non-protein coding transcript. (Os01t0757301-00)
Os08g0135900	1798.164	0.766837	0.001028		1425.261	0.168307	0.99986	Similar to Tryptophan synthase beta chain 1 (EC 4.2.1.20) (Orange pericarp 1) (Fragment). (Os08t0135900-01);Similar to Tryptophan synthase beta-subunit. (Os08t0135900-02);Similar to Tryptophan synthase beta-subunit. (Os08t0135900-03)
Os03g0251500	2373.723	0.683104	0.015568		1936.499	0.148176	0.99986	Similar to T-cell immune regulator 1 transcript variant 3 (Fragment). (Os03t0251500-01)
Os12g0640600	1985.79	0.802504	0.006432		1537.776	0.146793	0.99986	Carotenoid-cleavage dioxygenase (Os12t0640600-01)
Os01g0757200	208.3968	1.499969	0.000978		115.6904	0.14558	0.99986	GA 2-oxidase3, GA metabolism (Os01t0757200-01)
Os12g0190000	5815.379	0.854519	0.006621		4393.34	0.144522	0.99986	GDP-L-galactose phosphorylase, Ascorbate biosynthesis, Stress tolerance, Plant development (Os12t0190000-01);Similar to VTC2. (Os12t0190000-02)
Os03g0431200	161.1408	1.037458	0.015368		111.8948	0.144452	0.99986	Similar to N-acetyl-glutamate synthase. (Os03t0431200-00)
Os11g0618700	1132.521	0.939918	0.009512		815.6095	0.117178	0.99986	Protein of unknown function DUF594 domain containing protein. (Os11t0618700-01)
Os10g0159800	4104.74	0.896511	3.39E-08		2993.594	0.09784	0.99986	Alcohol dehydrogenase superfamily, zinc-

								containing protein. (Os10t0159800-01);Similar to Alcohol dehydrogenase 2. (Os10t0159800-03);Similar to Alcohol dehydrogenase 2. (Os10t0159800-04)
Os12g0437800	769.2863	1.358253	0.005632		448.7673	0.084992	0.99986	Similar to MPI. (Os12t0437800-01)
Os05g0545000	5537.048	0.557866	0.018729		4602.031	0.05358	0.99986	Similar to Phosphatidylinositol transfer-like protein IV. (Os05t0545000-01);Similar to Phosphatidylinositol transfer-like protein III. (Os05t0545000-02)
Os02g0803300	1184.217	0.733386	0.005288		912.8578	0.050924	0.99986	Epsin-like, N-terminal domain containing protein. (Os02t0803300-01)
Os04g0438600	529.754	-1.075683	0.003361		736.0044	0.044968	0.99986	Glycolipid transfer protein, GLTP domain containing protein. (Os04t0438600-01)
Os03g0128700	4901.81	0.588746	0.043413		4005.768	0.041751	0.99986	Calcium-dependent protein kinase, isoform 11 (EC 2.7.1.-) (CDPK 11). (Os03t0128700-01)
Os02g0285800	200.5841	0.909627	0.006416		141.9639	0.028785	0.99986	Similar to GTP-binding protein typA (Tyrosine phosphorylated protein A). (Os02t0285800-01);Similar to predicted protein. (Os02t0285800-02)
Os01g0780800	580.6384	0.669214	0.027821		455.9979	0.026208	0.99986	SAND domain-containing protein, Trithorax group factor, Transcriptional regulation of stress responsive genes (Os01t0780800-01);Similar to predicted protein. (Os01t0780800-02)
Os05g0161800	274.3431	1.382362	0.022373		154.1448	0.016879	0.99986	Calcium-activated (p)ppGpp synthetase, Ca²⁺-activated RelA-SpoT homolog 3 (Os05t0161800-01)
Os10g0550900	217.88	1.782673	6.03E-06		98.44933	-0.013092	0.99986	Proline dehydrogenase, Negative regulation of thermotolerance (Os10t0550900-01)
Os03g0784000	319.796	0.930987	0.000359		220.2974	-0.019048	0.99986	Similar to deoxyribodipyrimidine photolyase. (Os03t0784000-01);Similar to deoxyribodipyrimidine photolyase. (Os03t0784000-02);FAD dependent oxidoreductase family protein. (Os03t0784000-03)
Os12g0145100	3455.777	-0.670889	0.044344		4246.006	-0.023108	0.99986	Similar to Wali7 protein (Fragment). (Os12t0145100-01)
Os12g0145150	3182.517	-0.672141	0.0434		3908.949	-0.02507	0.99986	Hypothetical gene. (Os12t0145150-00)

Os02g0209300	150.4635	0.852817	0.044634		106.3785	-0.04669	0.99986	Hypothetical conserved gene. (Os02t0209300-01)
Os06g0581300	628.8662	0.603502	0.014562		492.8906	-0.060628	0.99986	Hypothetical conserved gene. (Os06t0581300-01);Protein of unknown function DUF1475 family protein. (Os06t0581300-02)
Os01g0847700	3469.741	0.726963	0.006551		2563.384	-0.080407	0.99986	Aldo-keto reductase, Detoxification of reactive aldehydes, Abiotic stress tolerance (Os01t0847700-01)
Os11g0676800	308.3532	-0.83237	0.01182		383.7047	-0.110315	0.99986	Similar to NB-ARC domain containing protein. (Os11t0676800-00)
Os05g0161700	231.0649	1.43874	0.023507		120.6185	-0.11552	0.99986	Hypothetical protein. (Os05t0161700-01)
Os05g0415800	1437.832	-0.734715	0.010219		1727.231	-0.14005	0.99986	Similar to Cytochrome P450 CYP51H10. (Os05t0415800-01)
Os01g0696701	887.9008	0.745859	0.030852		635.6087	-0.150413	0.99986	Hypothetical protein. (Os01t0696701-00)
Os05g0595300	810.0499	-1.129392	1.34E-06		1062.692	-0.158636	0.99986	NRR alternative splicing variant, Regulation of root development in response to macronutrient deficiency (Os05t0595300-01);CCT domain-containing protein, Regulator of starch synthesis, Regulation of root development in response to macronutrient deficiency (Os05t0595300-02)
Os06g0178800	63.19855	-1.453754	0.00378		87.01549	-0.212129	0.99986	Hypothetical protein. (Os06t0178800-00)
Os06g0178700	64.52597	-1.493816	0.003021		89.16293	-0.223642	0.99986	Similar to Iron/ascorbate-dependent oxidoreductase. (Os06t0178700-01)
Os07g0604000	1273.029	-1.021141	0.00021		1588.92	-0.238125	0.99986	Similar to 6-phosphogluconolactonase-like protein. (Os07t0604000-01);Similar to 6-phosphogluconolactonase-like protein. (Os07t0604000-02)
Os01g0521600	327.6742	-0.963439	0.020006		403.3692	-0.240231	0.99986	Similar to NB-ARC domain containing protein, expressed. (Os01t0521600-00)
Os12g0594950	47.91554	-1.489867	0.028189		65.57154	-0.256058	0.99986	Hypothetical conserved gene. (Os12t0594950-01)
Os07g0531400	193.5514	-1.093382	0.035572		242.8882	-0.270189	0.99986	Similar to Peroxidase 27 precursor (EC 1.11.1.7) (Atperox P27) (PRXR7) (ATP12a). (Os07t0531400-01);Plant peroxidase domain containing protein. (Os07t0531400-02)
Os01g0711400	2499.597	-0.843407	0.022362		2952.62	-0.276954	0.99986	Similar to Victorin binding protein. (Os01t0711400-01)
Os09g0549450	2537.794	-0.760026	0.001996		2922.336	-0.292911	0.99986	NLP family transcriptional factor, Promotion of

								nitrogen use efficiency (NUE), Transactivation of nitrogen assimilation gene OsNiR (Os09t0549450-01)
Os08g0500800	1139.115	-0.582883	0.017399		1244.119	-0.310255	0.99986	WLM domain containing protein. (Os08t0500800-01)
Os11g0609500	484.6858	-0.880319	0.002772		570.6574	-0.317271	0.99986	Similar to Jacalin-like lectin domain containing protein. (Os11t0609500-01)
Os11g0670900	78.77166	-1.874318	0.022973		112.2464	-0.320359	0.99986	Conserved hypothetical protein. (Os11t0670900-01)
Os10g0506100	1132.693	-0.878088	0.000601		1331.048	-0.322252	0.99986	Heavy metal transport/detoxification protein domain containing protein. (Os10t0506100-01)
Os05g0429400	2110.871	-1.082103	0.028189		2596.707	-0.325739	0.99986	Phosphatidylinositol transfer protein-like, N-terminal domain containing protein. (Os05t0429400-01)
Os05g0337200	549.1798	-0.772014	0.025428		623.4037	-0.349112	0.99986	Helix-loop-helix DNA-binding domain containing protein. (Os05t0337200-01)
Os05g0407100	94.702	-1.089199	0.00494		115.9235	-0.352551	0.99986	Four F5 protein family protein. (Os05t0407100-01)
Os10g0345701	110.2201	-0.951487	0.034923		130.0065	-0.369979	0.99986	Hypothetical conserved gene. (Os10t0345701-01)
Os08g0416800	1083.619	-0.802595	0.002074		1227.03	-0.382125	0.99986	Conserved hypothetical protein. (Os08t0416800-01)
Os08g0495500	2517.105	-0.727814	0.00021		2791.956	-0.387149	0.90027	Kelch related domain containing protein. (Os08t0495500-01)
Os01g0719600	526.2307	-0.70079	0.022362		578.1163	-0.396401	0.99986	Heavy metal transport/detoxification protein domain containing protein. (Os01t0719600-01)
Os12g0431300	42.34651	-2.356488	0.007239		62.84564	-0.3976	0.99986	Six-bladed beta-propeller, TolB-like domain containing protein. (Os12t0431300-01)
Os11g0151500	1032.057	-1.184456	0.00238		1261.263	-0.422712	0.99986	Major facilitator superfamily protein. (Os11t0151500-01)
Os02g0782900	973.0263	-0.736289	0.022096		1070.306	-0.423564	0.973604	Conserved hypothetical protein. (Os02t0782900-01)
Os01g0605700	194.6364	-1.482442	0.001336		250.4002	-0.447951	0.99986	MtN3 and saliva related transmembrane protein family protein. (Os01t0605700-01)
Os04g0598800	187.523	-0.981598	0.012142		216.3294	-0.466497	0.99986	Similar to Wall-associated kinase-like protein. (Os04t0598800-01)
Os06g0115600	616.901	-0.923855	0.011682		701.9956	-0.466892	0.99986	Plant-specific protein with a short C-terminal coiled-coil domain and a functional NLS, Common symbiosis signaling (SYM) pathway (Os06t0115600-01)
Os09g0272900	935.0844	-1.033947	0.00261		1086.328	-0.483268	0.99986	Disease resistance protein domain containing protein. (Os09t0272900-01);Hypothetical conserved gene. (Os09t0272900-02)
Os05g0592300	608.2361	-0.778337	0.00108		663.8019	-0.487221	0.881556	Protein of unknown function DUF1637 family

								protein. (Os05t0592300-01);Similar to Cupin, RmlC-type. (Os05t0592300-02)
Os11g0104300	171.0992	-1.512551	0.029941		218.0238	-0.49909	0.99986	Substrate of the SCF^{D3} ubiquitination complex, Repressor of strigolactone (SL) signalling (Os11t0104300-01)
Os09g0311600	931.8429	-0.807395	0.000123		1020.382	-0.501601	0.99986	Similar to NBS-LRR type resistance protein (Fragment). (Os09t0311600-01)
Os02g0575000	504.8381	1.064649	0.003021		279.2303	-0.520173	0.99986	Conserved hypothetical protein. (Os02t0575000-01)
Os03g0329200	110.6316	-1.196215	0.025308		131.3741	-0.528327	0.99986	Nucleotide-binding, alpha-beta plait domain containing protein. (Os03t0329200-01);Similar to cDNA clone:J013002B05, full insert sequence. (Os03t0329200-02)
Os12g0568350	41498.86	0.008248	0.999758		35174.5	-0.543028	0.042509	Hypothetical gene. (Os12t0568350-01)
Os12g0568500	41489.64	-0.008471	0.999758		35312.85	-0.548888	0.040984	Metallothionein-like protein type 1. (Os12t0568500-01)
Os03g0368300	669.274	-1.204318	0.035493		787.5674	-0.567349	0.99986	Similar to Peroxidase 1. (Os03t0368300-01)
Os03g0427000	394.0106	-0.995599	0.009588		440.9801	-0.581629	0.970711	Similar to Circadian clock coupling factor ZGT. (Os03t0427000-01)
Os01g0878300	1483.499	-0.909303	0.001996		1624.779	-0.589188	0.50379	Protein kinase, core domain containing protein. (Os01t0878300-01);Hypothetical conserved gene. (Os01t0878300-02)
Os03g0103300	2046.532	-0.930839	0.000258		2252.439	-0.589383	0.878752	Hybrid proline- or glycine-rich protein, Controll of low-temperature germinability, Pre-harvest sprouting resistance (Os03t0103300-01)
Os03g0803500	226.5778	-1.207081	0.02855		264.2787	-0.606191	0.99986	Similar to Prolyl 4-hydroxylase alpha-1 subunit-like protein. (Os03t0803500-01)
Os06g0611900	132.5439	-1.171978	0.006113		153.0897	-0.614253	0.99986	Similar to Glycine decarboxylase P subunit. (Os06t0611900-01);Similar to Victorin binding protein. (Os06t0611900-02)
Os02g0168400	309.9884	-0.879877	0.000491		333.0651	-0.634384	0.450013	Hypothetical conserved gene. (Os02t0168400-01)
Os08g0380100	193.6783	-1.765298	0.020594		246.8695	-0.649888	0.99986	Similar to Polygalacturonase isoenzyme 1 beta subunit (Fragment). (Os08t0380100-01);Similar to Polygalacturonase isoenzyme 1 beta subunit (Fragment). (Os08t0380100-02)

Os02g0631100	99.47117	-1.122411	0.021335		112.194	-0.657446	0.99986	Photosystem II oxygen evolving complex protein PsbQ family protein. (Os02t0631100-01)
Os02g0208500	420.3918	-0.753079	0.00921		433.5707	-0.666264	0.066661	Conserved hypothetical protein. (Os02t0208500-01)
Os04g0518100	2411.893	-1.50767	0.017327		2924.937	-0.670826	0.99986	Phenylalanine ammonia-lyase. (Os04t0518100-01)
Os08g0203700	466.4239	-0.796655	0.028189		485.1945	-0.674353	0.99986	Protein kinase, core domain containing protein. (Os08t0203700-01);Similar to SHR5-receptor-like kinase (Fragment). (Os08t0203700-02)
Os05g0363533	161.3955	-1.139541	0.004194		181.2163	-0.691928	0.99986	Non-protein coding transcript. (Os05t0363533-00)
Os05g0363500	1000.511	-1.304686	0.000121		1159.222	-0.701274	0.638892	Hypothetical conserved gene. (Os05t0363500-01)
Os05g0140334	893.8656	0.324582	0.75793		639.5617	-0.741584	0.038756	Hypothetical protein. (Os05t0140334-00)
Os03g0159900	113.938	-1.347522	0.000491		131.7066	-0.742545	0.529143	Hypothetical conserved gene. (Os03t0159900-01);Hypothetical conserved gene. (Os03t0159900-02)
Os03g0273200	521.1081	-1.8843	0.020802		656.908	-0.756666	0.99986	Similar to Laccase (EC 1.10.3.2). (Os03t0273200-01);Similar to Laccase (EC 1.10.3.2). (Os03t0273200-02)
Os08g0124500	225.7852	-0.711914	0.03773		224.9064	-0.757848	0.048415	Similar to Resistance protein candidate (Fragment). (Os08t0124500-01);Similar to Resistance protein candidate (Fragment). (Os08t0124500-02)
Os08g0507702	27.14353	-2.56515	0.026828		37.19586	-0.761404	0.99986	Cytochrome P450 family protein. (Os08t0507702-01)
Os10g0481300	187.8144	-1.460623	0.001276		218.508	-0.800708	0.99986	Similar to Glutathione S-transferase, N-terminal domain containing protein, expressed. (Os10t0481300-01)
Os04g0635650	118.791	-1.367056	0.022754		134.507	-0.843602	0.688906	Non-protein coding transcript. (Os04t0635650-00)
Os04g0635100	142.1739	-1.499138	0.015922		164.5241	-0.854673	0.749163	Similar to H0315F07.12 protein. (Os04t0635100-00)
Os08g0373400	45.47449	-1.496541	0.044954		52.07302	-0.887314	0.99986	Conserved hypothetical protein. (Os08t0373400-00)
Os04g0635201	110.8191	-1.597997	0.022108		129.3784	-0.8901	0.903287	Non-protein coding transcript. (Os04t0635201-00)
Os02g0782550	40.7355	-3.050394	1.60E-06		55.82238	-0.936809	0.511732	Hypothetical protein. (Os02t0782550-00)
Os09g0548200	148.4856	-1.480026	0.0434		167.706	-0.939058	0.99986	Peptidoglycan-binding Lysin subgroup domain containing protein. (Os09t0548200-01)
Os02g0782500	41.23887	-3.070153	1.30E-06		56.51796	-0.942928	0.50379	Similar to Small heat stress protein class CIII. (Os02t0782500-01);Similar to 17.5 kDa class II heat

								shock protein. (Os02t0782500-02)
Os01g0217000	367.9313	-1.681926	0.037469		414.9485	-1.091207	0.99986	Protein of unknown function DUF231, plant domain containing protein. (Os01t0217000-01);Similar to Alpha galactosidase. (Os01t0217000-02)
Os04g0105400	182.541	-1.904713	0.044468		212.6789	-1.100596	0.99986	Similar to B0616E02-H0507E05.3 protein. (Os04t0105400-01)
Os01g0880850	145.1224	-2.469636	1.77E-05		178.0851	-1.188285	0.564647	Hypothetical protein. (Os01t0880850-00)
Os01g0226600	138.6312	-1.943465	0.037469		158.0781	-1.22712	0.99986	Similar to C4-dicarboxylate transporter/malic acid transport protein. (Os01t0226600-01);Similar to C4-dicarboxylate transporter/malic acid transport protein. (Os01t0226600-02)
Os01g0880800	148.393	-2.419596	0.000113		178.7673	-1.250275	0.569292	Similar to Acyl-[acyl-carrier-protein] desaturase, chloroplast precursor (EC 1.14.19.2) (Stearoyl-ACP desaturase). (Os01t0880800-01)
Os12g0495600	72.29434	-1.344935	0.008128		74.15691	-1.251411	0.807863	NB-ARC domain containing protein. (Os12t0495600-01)
Os01g0795050	998.0303	-0.245791	0.999758		772.5858	-1.265891	0.00056	Hypothetical gene. (Os01t0795050-01)
Os01g0843800	39.53344	-2.454582	0.017834		47.08179	-1.332748	0.99986	Similar to L-ascorbate oxidase. (Os01t0843800-00)
Os01g0795000	1593.858	-0.192999	0.999758		1193.077	-1.349451	0.001221	Subtilase. (Os01t0795000-01)
Os04g0567200	58.79878	-2.211937	0.000674		67.74961	-1.364064	0.282038	Uncharacterised protein family UPF0005 domain containing protein. (Os04t0567200-01)
Os05g0424300	47.41355	-2.24385	0.029941		54.72882	-1.369413	0.99986	Cytochrome P450 family protein. (Os05t0424300-01)
Os01g0126900	340.5562	-2.420559	0.006113		395.6786	-1.432002	0.99986	Multicopper oxidase (Os01t0126900-01)
Os03g0219100	195.0948	-2.687035	0.002221		231.2565	-1.469177	0.99986	Cellular retinaldehyde-binding/triple function, C-terminal domain containing protein. (Os03t0219100-01)
Os12g0472900	93.53642	-2.836479	7.71E-05		110.057	-1.590391	0.619175	Hypothetical protein. (Os12t0472900-00)
Os07g0565100	313.1866	-0.54736	0.77227		247.4976	-1.644833	9.69E-05	Similar to 40S ribosomal protein S11. (Os07t0565100-00)
Os01g0318400	14.21258	-3.251638	0.006416		16.88819	-1.749834	0.849414	Conserved hypothetical protein. (Os01t0318400-00)
Os04g0587400	21.29822	-3.261235	0.032607		25.18764	-1.759851	0.99986	Protein of unknown function DUF250 domain containing protein. (Os04t0587400-01)
Os03g0257600	90.53777	-2.583376	0.012291		100.2009	-1.819168	0.565095	Hypothetical conserved gene. (Os03t0257600-01)
Os12g0472800	226.0648	-3.170641	0.000328		258.752	-1.919698	0.625326	B repeat unit of collagen binding surface protein

								(cna) containing protein. (Os12t0472800-01)
Os02g0665300	28.51481	-3.849629	0.010578		31.35657	-2.579088	0.666681	Peptidase S8, subtilisin-related domain containing protein. (Os02t0665300-01);Peptidase S8, subtilisin-related domain containing protein. (Os02t0665300-02)
Os03g0226200	71.52727	0.077959	0.999758		38.31174	-3.459743	0.000234	Non-symbiotic hemoglobin, Phytoglobin, Response to low mineral nutrients and abiotic stresses (Os03t0226200-01)
