# Supplementary material for: Effects of LPS from Rhodobacter sphaeroides, a Purple Non-Sulfur Bacterium (PNSB), on the Gene Expression of Rice Root
Source: Microorganisms. 2023 Jun 28;11(7):1676. doi: 10.3390/microorganisms11071676 (PMC10383378; doi:10.3390/microorganisms11071676)
Supplement: Supplementary file 1 [file microorganisms-11-01676-s001.zip › Table S2.pdf]

Table S2 List of 36 JA signaling pathway related genes

LPS				Dead cells			
GeneID	baseMean	logFC	padj	baseMean	logFC	padj	Description
Os03g0180900	331.6668	3.088268	9.08E-24	198.588	2.21663	1.03E-10	Jasmonate ZIM-domain containing protein, Transcriptional repressor of JA signaling, Regulation of phosphate starvation responses, Pi homeostasis (Os03t0180900-01)
Os05g0586200	5241.215	2.528499	6.41E-23	2774.058	1.35187	2.88E-06	Gretchen hagen 3 (GH3) family protein, Phytochrome, Jasmonyl-L-isoleucine synthase, Jasmonate signaling, Wound response, Response to blast infection (Os05t0586200-01)
Os09g0439200	327.6254	1.9726	2.56E-17	225.7917	1.242014	9.78E-05	Jasmonate ZIM-domain protein, Jasmonate-induced resistance to bacterial blight, Repressor of jasmonic acid signaling (Os09t0439200-01)
Os11g0684000	133.8181	3.246165	3.99E-13	56.33285	1.756915	0.003497	Myb transcription factor, Jasmonate-dependent defense response, Negative regulation of bacterial blight resistance (Os11t0684000-01)
Os10g0391400	32.8428	1.871914	0.246463	53.39051	2.702588	0.008779	Jasmonate ZIM-domain (JAZ) protein, TIFY family protein, Negative regulation of JA signal transduction pathway, Activation of hypersensitive cell death (Os10t0391400-01)
Os07g0153000	12.12568	3.511223	0.027326	12.04041	3.484357	0.036289	A member of the JASMONATE ZIM-domain (JAZ) family (Os07t0153000-01)
Os03g0402800	2106.674	1.712575	1.06E-11	1420.237	0.894661	0.038213	TIFY family protein, JASMONATE-ZIM domain (JAZ) protein, JA signaling, Regulation of spikelet development (Os03t0402800-01);Similar to ZIM motif family protein, expressed. (Os03t0402800-02)
Os09g0401300	2417.472	1.452998	2.13E-19	1618.989	0.568303	0.040984	A member of the JASMONATE ZIM-domain (JAZ)

							family, Negative modulation of JA signaling and antiviral defense (Os09t0401300-01);Similar to ZIM motif family protein. (Os09t0401300-02);Similar to ZIM motif family protein. (Os09t0401300-03)
Os04g0301500	35.6641	8.635412	1.38E-08	2.722173	4.910923	0.733147	Basic helix-loop-helix transcription activator, Control of disease resistance, Regulation of salicylic acid (SA) and jasmonic acid (JA) signaling (Os04t0301500-01)
Os01g0221100	14.2594	-1.327714	0.836536	14.59437	-1.239343	0.99986	Jasmonyl-L-isoleucine synthase, Wound response, Jasmonic acid signalling (Os01t0221100-01);GH3 auxin-responsive promoter family protein. (Os01t0221100-02)
Os03g0265500	967.4581	0.191417	0.999758	909.7759	-0.002955	0.99986	COI1 homolog, Jasmonate signaling (Os03t0265500-01);Similar to Coronatine-insensitive 1. (Os03t0265500-02)
Os04g0653000	6158.263	0.669641	0.013931	5386.171	0.318287	0.99986	Jasmonic acid (JA) signalling repressor, Regulation of spikelet development (Os04t0653000-01)
Os05g0143600	0.473559	0.871852	0.999758	0.891452	2.04278	0.99986	Similar to Jasmonate-induced protein. (Os05t0143600-01)
Os10g0132300	2538.243	-0.068078	0.999758	2638.813	0.019928	0.99986	Similar to Jasmonate-induced protein. (Os10t0132300-01)
Os11g0446000	190.4762	-1.049146	0.719468	187.7582	-1.14172	0.99986	Conserved hypothetical protein. (Os11t0446000-01);Nuclear protein homologous to the regulatory domain of the R/B-like bHLH transcription factors, Regulation of jasmonate-induced gene expression and root cell elongation (Os11t0446000-02)
Os12g0144200	6.203599	2.757408	0.334296	5.02001	2.386029	0.99986	Similar to Jasmonate-induced protein. (Os12t0144200-00)
Os12g0198700	12.75082	0.067676	0.999758	14.85312	0.456647	0.99986	Similar to Jasmonate-induced protein. (Os12t0198700-01)
Os03g0119966	129.6068	0.101829	0.999758	126.889	0.014312	0.99986	NAC-domain protein, Resistance to rice dwarf virus, Regulator of

							jasmonate (JA) signaling (Os03t0119966-01)
Os02g0559400	203.9827	0.030942	0.999758	214.3766	0.147087	0.99986	12-oxophytodienoic acid reductase (EC:1.3.1.-), Jasmonic acid (JA)-mediated defense signaling (Os02t0559400-01);Similar to 12-oxo-phytodienoic acid reductase5. (Os02t0559400-02)
Os06g0216300	5143.767	0.468807	0.270774	4817.961	0.280539	0.99986	Similar to 12-oxophytodienoic acid reductase. (Os06t0216300-01);12-oxophytodienoic acid reductase 1, Jasmonic acid (JA) biosynthesis, Defense response (Os06t0216300-02)
Os08g0459600	2389.871	0.448955	0.228751	2230.917	0.251335	0.99986	12-oxophytodienoate reductase (EC:1.3.1.42), Jasmonic acid (JA) biosynthesis (Os08t0459600-01)
Os02g0218700	0.488675	0.871852	0.999758	0.173578	-0.910699	0.99986	Allene oxide synthase (CYP74A3), Fatty acid 9-/13-hydroperoxide lyase (CYP74C), Biosynthesis of jasmonic acid (JA), Plant defense (Os02t0218700-01)
Os02g0218800	974.2635	-0.573114	0.989443	948.499	-0.695472	0.99986	Allene oxide synthase (CYP74A4), Fatty acid 9-/13-hydroperoxide lyase (CYP74C), Biosynthesis of jasmonic acid (JA), Plant defense (Os02t0218800-01)
Os03g0767000	166.6354	1.067698	0.560411	118.5652	0.250317	0.99986	Allene oxide synthase (CYP74A1), Biosynthesis of jasmonic acid (JA) (Os03t0767000-01)
Os05g0375400	5227.694	-1.127354	0.484538	5071.326	-1.301653	0.99986	Endo-(1,3;1,4)-β-glucanase, "Response to wounding, methyl jasmonate, abscisicacid and ethephon" (Os05t0375400-01)
Os03g0300400	9281.074	-0.054414	0.999758	9995.171	0.132007	0.99986	Jasmonate inducible pathogenesis-related class 10 protein, Self-defense against biotic and abiotic stresses (Os03t0300400-01)
Os03g0225900	9.766175	1.038629	0.996501	8.744091	0.773251	0.99986	Allene oxide synthase (CYP74A2), Biosynthesis of jasmonic acid (JA)

							(Os03t0225900-01);Allene oxide synthase. (Os03t0225900-02)
Os07g0247100	166.3662	0.048609	0.999758	161.1097	-0.069787	0.99986	Chloroplast-localised putative leucine carboxyl methyltransferase (LCMT), O-methyltransferase, Regulation of jasmonate (JA)- and brassinosteroid (BR)-mediated growth and defence responses, Leaf senescence, heading date and grain production via melatonin biosynthesis (Os07t0247100-01)
Os01g0853400	805.5175	-0.421146	0.999758	886.3519	-0.137105	0.99986	Component of the SCF E3 ubiquitin ligase complex, Jasmonate-regulated defense responses, Promoting leaf senescence (Os01t0853400-01)
Os01g0900400	0.948696	-3.336362	0.999758	2.454627	0.629917	0.99986	Putative triacylglycerol (TAG) lipase, Phospholipase A1, Specification of empty-glume identity, Regulation of spikelet development, Endogenous jasmonic acid (JA) biosynthesis (Os01t0900400-01)
Os03g0438100	2675.702	0.952432	0.289824	2182.055	0.461152	0.99986	Allene oxide cyclase, Biosynthesis of jasmonic acid, Defence response (Os03t0438100-01);Similar to Allene oxide cyclase precursor (EC 5.3.99.6) (Allene oxide cylase). (Os03t0438100-02)
Os05g0102000	3.354277	1.302677	0.999758	3.326359	1.271619	0.99986	Jasmonic acid carboxyl methyltransferase, Regulation of plant development and herbivory-induced defense response, Resistance to brown planthopper (BPH) (Os05t0102000-01)
Os05g0449500	983.6981	-0.882955	0.398254	1058.755	-0.624761	0.99986	Component of the SCF E3 ubiquitin ligase complex, Jasmonate-regulated defense responses, Regulation of leaf senescence (Os05t0449500-01)

Os06g0551400	2227.199	0.087112	0.999758	2153.191	-0.033424	0.99986	Ras-related small GTP-binding protein, Regulation of vesicular trafficking from trans-Golgi network to plasma membrane or vacuole, Jasmonic acid (JA)-mediated defense signaling (Os06t0551400-01)
Os08g0500100	1760.003	0.060103	0.999758	1803.824	0.10587	0.99986	Peroxisomal targeting sequence1 (PTS1) receptor protein, Type I peroxisomal targeting signal (PTS1) receptor, Regulation of spikelet development, Modulation of jasmonic acid biosynthesis, Antifungal activity (Os08t0500100-01);Peroxisomal targeting sequence1 (PTS1) receptor protein, Type I peroxisomal targeting signal (PTS1) receptor, Regulation of spikelet development, Modulation of jasmonic acid biosynthesis, Antifungal activity (Os08t0500100-02)
Os10g0575000	231.9871	-0.264034	0.999758	232.7841	-0.275863	0.99986	JAZ-interacting transcription factor, Positive regulator of jasmonate (JA) signaling pathway, Bacterial blight resistance, Spikelet development (Os10t0575000-01)
