# Supplementary material for: Effects of LPS from Rhodobacter sphaeroides, a Purple Non-Sulfur Bacterium (PNSB), on the Gene Expression of Rice Root
Source: Microorganisms. 2023 Jun 28;11(7):1676. doi: 10.3390/microorganisms11071676 (PMC10383378; doi:10.3390/microorganisms11071676)
Supplement: Supplementary file 1 [file microorganisms-11-01676-s001.zip › Table S3.pdf]

Table S3 List of 21 chalcone synthase genes (Figure 4)

LPS				Dead cells			
GeneID	baseMean	logFC	padj	baseMean	logFC	padj	Description
Os05g0212600	29.29402	2.410994	0.419049	32.34188	2.574478	0.019425	Chalcone/stilbene synthase, N-terminal domain containing protein. (Os05t0212600-00)
Os10g0168300	21.23668	2.373493	0.346504	28.21588	2.843561	0.001305	Similar to Chalcone and stilbene synthases, N-terminal domain containing protein. (Os10t0168300-00)
Os10g0168500	7.804006	3.886519	0.136665	8.924321	4.092004	0.071341	Similar to Chalcone and stilbene synthases, N-terminal domain containing protein. (Os10t0168500-00)
Os10g0177300	20.56308	5.378024	0.006105	19.48063	5.280179	0.000234	Similar to Chalcone and stilbene synthases, N-terminal domain containing protein. (Os10t0177300-00)
Os11g0529036	5.914962	4.123683	0.400558	9.592993	4.830888	0.032533	Similar to Chalcone and stilbene synthases, N-terminal domain containing protein. (Os11t0529036-00)
Os11g0529100	11.7402	3.229448	0.484836	15.45315	3.649044	0.009083	Similar to Chalcone and stilbene synthases, N-terminal domain containing protein. (Os11t0529100-00)
Os11g0529125	1.963383	4.447775	0.770926	4.09374	5.498691	0.37864	Similar to Chalcone and stilbene synthases, N-terminal domain containing protein. (Os11t0529125-00)
Os11g0529900	78.55086	-0.371614	0.999758	70.4007	-0.789074	0.99986	Chalcone/stilbene synthase, N-terminal domain containing protein. (Os11t0529900-00)
Os04g0103900	4.119857	3.564309	0.487887	0.490591	-0.812004	0.99986	Similar to Chalcone synthase. (Os04t0103900-01)
Os05g0212900	351.6929	1.788267	0.184124	404.54	2.031264	9.78E-05	Similar to Chalcone synthase J (EC 2.3.1.74) (Naringenin-chalcone synthase J). (Os05t0212900-00)
Os05g0213100	215.9852	1.741442	0.353929	264.0679	2.096895	0.072893	Similar to Chalcone and stilbene synthases, N-terminal domain containing protein. (Os05t0213100-00)
Os07g0214900	15.73968	0.649187	0.999758	16.95568	0.815933	0.99986	Chalcone synthase,

							Sakuranetin biosynthesis under UV stress conditions (Os07t0214900-01)
Os07g0271500	1.505223	0.4571	0.999758	0.821158	-1.802872	0.99986	Similar to Chalcone synthase. (Os07t0271500-01)
Os07g0525900	0.517925	1.036191	0.999758	0.157576	-0.910699	0.99986	Similar to Chalcone synthase. (Os07t0525900-00)
Os10g0162856	419.6071	1.351509	0.337794	423.2235	1.354421	0.01565	Similar to Chalcone and stilbene synthases, N-terminal domain containing protein. (Os10t0162856-00)
Os02g0778500	199.0228	-0.02007	0.999758	220.4712	0.241649	0.99986	Chalcone isomerase domain containing protein. (Os02t0778500-01)
Os06g0203600	241.4538	0.36881	0.83351	238.8641	0.319295	0.99986	Chalcone isomerase domain containing protein. (Os06t0203600-01)
Os07g0571600	53.57363	-0.183078	0.999758	59.0907	0.080611	0.99986	Chalcone isomerase domain containing protein. (Os07t0571600-00)
Os07g0411300	14.70538	1.266973	0.691628	13.08771	1.010168	0.99986	Polyketide synthase, Pollen exine and Ubisch body patterning, Pollen wall formation (Os07t0411300-01)
Os10g0158400	430.8074	1.152041	0.464978	418.8224	1.077063	0.137006	Type III polyketide synthase (Os10t0158400-01)
Os10g0167900	142.3608	3.308983	0.369027	141.2312	3.285438	7.17E-05	Type III polyketide synthase (Os10t0167900-01)
