# Supplementary material for: Effects of LPS from Rhodobacter sphaeroides, a Purple Non-Sulfur Bacterium (PNSB), on the Gene Expression of Rice Root
Source: Microorganisms. 2023 Jun 28;11(7):1676. doi: 10.3390/microorganisms11071676 (PMC10383378; doi:10.3390/microorganisms11071676)
Supplement: Supplementary file 1 [file microorganisms-11-01676-s001.zip › Table S4.pdf]

Table S4 List of 14 genes coding ROS/RNS-generating enzymes (Figure 5), including one NADH oxidase, 9 NADPH oxidase and 4 nitric oxide synthase genes

LPS				Dead cells			
GeneID	baseMean	logFC	padj	baseMean	logFC	padj	Description
Os01g0360200	5926.35	0.411546	0.583766	5431.719	0.160031	0.99986	NADPH oxidase, Disease resistance (Os01t0360200-01);Similar to Respiratory burst oxidase homolog. (Os01t0360200-03)
Os01g0370000	1007.306	1.139927	0.048896	1113.616	1.330914	0.000765	NADH:flavin oxidoreductase/NADH oxidase, N-terminal domain containing protein. (Os01t0370000-01)
Os01g0734200	1107.331	-0.564871	0.707804	1189.152	-0.346035	0.99986	Plasma membrane NADPH oxidase, Developmental regulation, Drought-stress response (Os01t0734200-01)
Os01g0784800	5575.759	-0.152284	0.999758	5891.946	-0.012816	0.99986	Flavodoxin/nitric oxide synthase domain containing protein. (Os01t0784800-01)
Os02g0104700	93.52522	-0.407993	0.962912	103.6077	-0.109775	0.99986	Nitric oxide synthase, Chloroplast ribosome biogenesis and/or translation, Regulation of biosynthesis of chloroplast proteins at lower temperature (Os02t0104700-01);Similar to Nitric oxide associated 1. (Os02t0104700-02)
Os03g0279900	501.9288	0.196645	0.999758	473.1344	0.009353	0.99986	Nitric oxide synthase-interacting domain containing protein. (Os03t0279900-01)
Os03g0775300	186.0569	-0.407513	0.960804	192.0499	-0.329625	0.99986	Flavodoxin/nitric oxide synthase domain containing protein. (Os03t0775300-01)
Os05g0528000	1504.079	-0.240178	0.999758	1588.656	-0.095724	0.99986	Respiratory burst oxidase homolog protein, Reactive oxygen species formation (Os05t0528000-01)
Os12g0541300	37.57515	-1.251504	0.62038	34.03056	-1.854449	0.750034	NADPH oxidase, Respiratory burst oxidase homolog, Ethylene-induced aerenchyma formation in roots under oxygen-deficient conditions (Os12t0541300-01)
Os01g0835500	809.8171	-0.17	0.999758	811.6179	-0.187867	0.99986	NADPH oxidase (Int. J. Mol. Sci. 2013, 14, 9440-9458; doi:10.3390/ijms14059440)
Os05g0465800	8.33076	0.264029	0.999758	8.966565	0.445305	0.99986	NADPH oxidase (Int. J. Mol. Sci. 2013, 14, 9440-9458; doi:10.3390/ijms14059440)
Os08g0453700	317.9656	-0.722887	0.729889	310.6306	-0.841971	0.99986	NADPH oxidase (Int. J. Mol. Sci. 2013, 14, 9440-9458; doi:10.3390/ijms14059440)
Os09g0438000	525.1351	-0.806681	0.460604	598.3608	-0.362979	0.99986	NADPH oxidase (Int. J. Mol. Sci. 2013, 14, 9440-9458; doi:10.3390/ijms14059440)
Os11g0537400	87.26725	-0.838328	0.868004	84.05682	-1.025326	0.99986	NADPH oxidase (Int. J. Mol. Sci. 2013, 14, 9440-9458; doi:10.3390/ijms14059440)
