# Supplementary material for: Effects of LPS from Rhodobacter sphaeroides, a Purple Non-Sulfur Bacterium (PNSB), on the Gene Expression of Rice Root
Source: Microorganisms. 2023 Jun 28;11(7):1676. doi: 10.3390/microorganisms11071676 (PMC10383378; doi:10.3390/microorganisms11071676)
Supplement: Supplementary file 1 [file microorganisms-11-01676-s001.zip › Table S5.pdf]

Table S5 List of 57 glutathione S-transferase genes (Figure 6)

LPS				Dead cell			
GeneID	baseMean	logFC	padj	baseMean	logFC	padj	Description
Os01g0353400	17.6547642	-0.6749816	0.9997583	14.9807217	-1.4251431	0.86812684	Glutathione S-transferase, Tolerance to salinity and oxidative stress, ROS-scavenging activity (Os01t0353400-01)
Os01g0369700	8246.97786	0.00878457	0.9997583	8560.40646	0.09135341	0.99985955	Similar to Glutathione S-transferase GST 8 (EC 2.5.1.18). (Os01t0369700-01);Similar to Glutathione S-transferase GST 8 (EC 2.5.1.18). (Os01t0369700-03)
Os01g0371500	8306.16306	1.00593351	0.00247544	7726.82679	0.82752692	0.05213049	Similar to Glutathione-S-transferase 19E50. (Os01t0371500-01)
Os01g0372400	0.15931913	-0.8928971	0.9997583	0.85983787	1.97792521	0.99985955	Glutathione S-transferase, C-terminal-like domain containing protein. (Os01t0372400-01)
Os01g0374000	1.51563337	-0.8708423	0.9997583	6.09889469	2.37257282	0.97468288	Similar to Glutathione S-transferase I (EC 2.5.1.18) (GST-I) (GST-29) (GST class- phi). (Os01t0374000-01)
Os01g0692000	33.7868084	-0.7422364	0.79154664	48.4464145	0.35358115	0.99985955	Similar to Glutathione S-transferase GSTU6. (Os01t0692000-01)
Os01g0692100	105.182467	-0.579467	0.58098864	117.301405	-0.2399701	0.99985955	Glutathione S-transferase, C-terminal-like domain containing protein. (Os01t0692100-01)
Os01g0764000	93722.6658	-0.0373546	0.9997583	99009.7613	0.09560298	0.99985955	Similar to Glutathione S-transferase I (EC 2.5.1.18) (GST-I) (GST-29) (GST class- phi). (Os01t0764000-01);Similar to Glutathione s-transferase ii (Fragment). (Os01t0764000-02);Similar to Glutathione S-transferase I (EC 2.5.1.18) (GST-I) (GST-29) (GST class- phi). (Os01t0764000-03)

Os01g0949700	316.512158	0.0765036	0.9997583	308.216701	-0.021306	0.99985955	Glutathione S-transferase, C-terminal domain containing protein. (Os01t0949700-01)
Os01g0949750	718.290303	-0.2544097	0.9997583	714.677849	-0.296854	0.99985955	Similar to Glutathione S-transferase GST 28 (Fragment). (Os01t0949750-00)
Os01g0949800	15.3790267	0.2821488	0.9997583	15.2603185	0.23629268	0.99985955	Similar to Glutathione S-transferase GST 28 (EC 2.5.1.18) (Fragment). (Os01t0949800-01)
Os01g0950000	215.266094	1.33918976	0.176127	242.377972	1.55904993	0.04625054	Similar to Glutathione S-transferase GST 28 (EC 2.5.1.18) (Fragment). (Os01t0950000-01)
Os02g0319300	1100.30492	0.07884794	0.9997583	1085.70393	0.01783257	0.99985955	Glutathione S-transferase, C-terminal-like domain containing protein. (Os02t0319300-01)
Os02g0564000	781.545529	-0.0173567	0.9997583	785.541736	-0.0260339	0.99985955	Similar to Glutathione S-transferase. (Os02t0564000-01)
Os02g0814800	4.4945036	-0.5738402	0.9997583	4.28346893	-0.8057472	0.99985955	Glutathione S-transferase, C-terminal-like domain containing protein. (Os02t0814800-01)
Os03g0134900	1669.03495	0.25049082	0.9997583	1595.50296	0.108368	0.99985955	Similar to Glutathione S-transferase GSTF14 (Fragment). (Os03t0134900-01)
Os03g0135100	1550.86875	-0.0271579	0.9997583	1609.28551	0.05519604	0.99985955	Glutathione S-transferase GSTF15. (Os03t0135100-01)
Os03g0135200	794.267907	-0.2098556	0.9997583	845.507851	-0.046383	0.99985955	Similar to Glutathione S-transferase GST 9 (EC 2.5.1.18). (Os03t0135200-01)
Os03g0135300	260.847786	0.16708404	0.9997583	253.547579	0.06519898	0.99985955	Similar to Glutathione S-transferase GST 10 (EC 2.5.1.18). (Os03t0135300-01)
Os03g0283100	4690.98884	0.07605347	0.9997583	4656.66127	0.03200748	0.99985955	Lambda class glutathione S-transferase, Maize In2-1 homologue, Detoxification of some chemical compounds (Os03t0283100-01);Similar to In2-1 protein. (Os03t0283100-02)

Os03g0595600	0.52619954	1.00846536	0.9997583	0.49766447	0.89255096	0.99985955	Similar to Glutathione S-transferase, N-terminal domain containing protein, expressed. (Os03t0595600-00)
Os04g0435500	273.785572	-0.2495923	0.9997583	305.154971	0.05243199	0.99985955	Glutathione S-transferase, C-terminal-like domain containing protein. (Os04t0435500-01);Glutathione S-transferase, C-terminal-like domain containing protein. (Os04t0435500-02)
Os06g0168000	685.926852	0.02932851	0.9997583	675.697046	-0.0382975	0.99985955	Glutathione S-transferase, C-terminal-like domain containing protein. (Os06t0168000-01)
Os07g0168300	181.42289	0.53571491	0.90057491	170.026934	0.35664725	0.99985955	Similar to Glutathione S-transferase GSTU6. (Os07t0168300-01)
Os07g0468100	148.219424	0.56857979	0.9997583	157.220792	0.694192	0.99985955	Similar to Glutathione S-transferase GST 19 (EC 2.5.1.18). (Os07t0468100-01);Similar to Glutathione S-transferase GST 19 (EC 2.5.1.18). (Os07t0468100-02)
Os08g0293400	6.21542201	-0.8736342	0.9997583	5.95977587	-1.0667403	0.99985955	Similar to Glutathione S-transferase. (Os08t0293400-01)
Os08g0550500	273.194198	-0.1084726	0.9997583	271.428301	-0.1519864	0.99985955	Similar to Glutathione s-transferase (Fragment). (Os08t0550500-01)
Os09g0367700	2300.47192	-0.3051138	0.9997583	2441.25283	-0.1434843	0.99985955	Tau class glutathione S-transferase, Tolerance against sheath blight disease (Os09t0367700-01)
Os10g0368100	55.6086534	-0.7931328	0.60748446	58.4685739	-0.6385016	0.99985955	Similar to Glutathione S-transferase GSTU35 (Fragment). (Os10t0368100-01)
Os10g0481300	187.81437	-1.4606235	0.00127622	218.507984	-0.8007085	0.99985955	Similar to Glutathione S-transferase, N-terminal domain containing protein, expressed. (Os10t0481300-01)

Os10g0525400	111.994981	-0.3747168	0.9997583	128.917751	0.03360821	0.99985955	Similar to Glutathione S-transferase GSTU31 (Fragment). (Os10t0525400-01);Similar to glutathione S-transferase GST 34. (Os10t0525400-03)
Os10g0525500	221.61041	-0.6281091	0.30392287	224.13463	-0.6174553	0.50379024	Similar to glutathione transferase31. (Os10t0525500-01)
Os10g0525800	216.686015	-0.6175566	0.20516778	252.924976	-0.1319361	0.99985955	Similar to Glutathione S-transferase GSTU31 (Fragment). (Os10t0525800-00)
Os10g0527400	523.631541	-0.2036716	0.9997583	473.77792	-0.5627408	0.43960551	Similar to Tau class GST protein 3. (Os10t0527400-01)
Os10g0527601	486.873559	-0.6076473	0.56268435	461.359893	-0.8449023	0.90026975	Glutathione S-transferase, C-terminal-like domain containing protein. (Os10t0527601-00)
Os10g0527800	2074.60424	0.0069774	0.9997583	1862.34388	-0.3485036	0.99985955	Glutathione S-transferase, Regulation of leaf senescence (Os10t0527800-01)
Os10g0528100	19.4217116	0.55592343	0.9997583	24.7749254	1.09688663	0.99985955	Similar to Glutathione S-transferase GST 42 (EC 2.5.1.18) (Fragment). (Os10t0528100-01)
Os10g0528200	95.4509961	0.1816307	0.9997583	79.7640038	-0.3710577	0.99985955	Similar to Glutathione S-transferase TSI-1 (EC 2.5.1.18) (Glutathione S-transferase 1). (Os10t0528200-01)
Os10g0528300	15121.8564	-0.5032463	0.59812793	15905.8129	-0.3605093	0.99985955	Tau class GST protein 4. (Os10t0528300-01)
Os10g0528400	4087.5061	0.19696724	0.9997583	3784.36365	-0.042806	0.99985955	Glutathione S-transferase, C-terminal-like domain containing protein. (Os10t0528400-01)
Os10g0528900	17.2298628	0.27428104	0.9997583	17.1163931	0.2334173	0.99985955	Similar to Tau class GST protein 3. (Os10t0528900-01)
Os10g0529300	3134.47186	0.35653882	0.49082945	3447.95174	0.5729982	0.99985955	Similar to Tau class GST protein 3. (Os10t0529300-01)
Os10g0529400	1871.0056	0.29131626	0.77936455	1833.88101	0.21639641	0.99985955	Similar to Glutathione S-transferase GSTU6. (Os10t0529400-01)

Os10g0529500	536.243081	0.08311505	0.9997583	485.411864	-0.2365997	0.99985955	Tau class glutathione S-transferase, Heavy metal and drought stress tolerance (Os10t0529500-01)
Os10g0529700	25.626413	-0.7856339	0.94275992	25.1115052	-0.9022717	0.99985955	Similar to glutathione transferase35. (Os10t0529700-01)
Os10g0529800	484.8533	-0.1779624	0.9997583	457.403088	-0.3872773	0.99985955	Similar to Tau class GST protein 4. (Os10t0529800-00)
Os10g0530200	5378.92187	1.41121582	0.05811231	6406.93234	1.73302886	9.69E-05	Similar to Glutathione S-transferase TSI-1 (EC 2.5.1.18) (Glutathione S-transferase 1). (Os10t0530200-01)
Os10g0530400	106.323752	0.40503076	0.9997583	77.5407453	-0.5504403	0.99985955	Similar to Glutathione-S-transferase Cla47. (Os10t0530400-01)
Os10g0530500	2.32109565	2.6606507	0.97414645	1.24795385	1.56450567	0.99985955	Similar to Glutathione-S-transferase Cla47. (Os10t0530500-01)
Os10g0530600	63.8003888	0.42178721	0.98327061	56.1177503	0.06307373	0.99985955	Similar to Glutathione S-transferase GST 20 (EC 2.5.1.18). (Os10t0530600-01)
Os10g0530900	2686.73418	1.33054807	9.50E-05	2670.39228	1.30294398	0.56509499	Similar to Glutathione S-transferase GST 30 (EC 2.5.1.18). (Os10t0530900-01);Similar to cDNA clone:006-203-F02, full insert sequence. (Os10t0530900-03)
Os10g0531300	642.610369	0.07216628	0.9997583	650.126024	0.08628842	0.99985955	Similar to Glutathione transferase (EC 2.5.1.18). (Os10t0531300-01)
Os10g0531400	650.523052	0.06944562	0.9997583	666.932798	0.12050287	0.99985955	Glutathione S-transferase GST 30 (EC 2.5.1.18). (Os10t0531400-01)
Os10g0543800	469.568089	-0.392123	0.9997583	459.986875	-0.4837871	0.99985955	Similar to Glutathione S-transferase GST 11 (EC 2.5.1.18). (Os10t0543800-00)
Os11g0245100	85.7082185	0.09235883	0.9997583	79.5410926	-0.1500874	0.99985955	Similar to Glutathione S-transferase 2. (Os11t0245100-01)
Os12g0123200	58.0386091	-0.182632	0.9997583	60.657312	-0.0754561	0.99985955	Glutathione S-transferase GST 7 (EC 2.5.1.18). (Os12t0123200-01)
